# MSC1 Cells Suppress Colorectal Cancer Cell Growth via Metabolic Reprogramming, Laminin–Integrin Adhesion Signaling, Oxidative Stress Resistance, and a Tumor-Suppressive Secretome

**DOI:** 10.3390/biomedicines13061503

**Published:** 2025-06-19

**Authors:** Panagiota-Angeliki Galliou, Niti Argyri, Papaioannou Maria, George Koliakos, Nikolaos A. Papanikolaou

**Affiliations:** Laboratory of Biological Chemistry, School of Medicine, Aristotle University of Thessaloniki, 54124 Thessaloniki, Greece; argyrw.niti@gmail.com (N.A.); mpapaioannou@auth.gr (P.M.); gkoliako@auth.gr (G.K.); papnik@auth.gr (N.A.P.)

**Keywords:** MSC1 phenotype, mesenchymal stem cells (MSCs), colorectal cancer (CRC), TLR4 signaling, lipopolysaccharide (LPS), protein-protein interaction (PPI) network, metabolic reprogramming, tumor-suppressive secretome, Wharton’s jelly mesenchymal stem cells (WJ-MSCs), ECM adhesion

## Abstract

**Background/Objectives:** Mesenchymal stem cells (MSCs) possess immunomodulatory properties, tumor-homing, and low immunogenicity, making them attractive for cell-based cancer therapies, but their role in colorectal cancer (CRC) remains controversial. The MSC1 phenotype, a pro-inflammatory, tumor-suppressive state induced by short-term, low-dose LPS activation via TLR4, has shown therapeutic promise but remains poorly characterized in CRC. We aimed to elucidate MSC1’s tumor-suppressive mechanisms and validate its activity against CRC cells using an integrated bioinformatics and in vitro approach. **Methods:** We constructed a high-confidence protein-protein interaction (PPI) network in Wharton’s jelly-derived MSCs (WJ-MSCs) following TLR4 activation to uncover enriched signaling pathways, transcriptional regulators, and secreted factors. Functional and transcriptional enrichment analyses pinpointed key mechanisms. We then co-cultured MSC1 cells with CRC cells to assess effects on proliferation and metabolism. **Results:** Network analysis revealed six tumor-suppressive mechanisms of MSC1 cells: (i) Metabolic reprogramming via enhanced glucose and lipid uptake, phosphoinositide signaling, and membrane/protein recycling, (ii) Robust antioxidant defenses, including SOS signaling and system xc⁻, (iii) Extracellular matrix stabilization and laminin-111–integrin-mediated adhesion, (iv) Secretome with direct anti-cancer effects, (v) Regulation of survival and cancer-associated fibroblasts (CAFs) formation inhibition through balanced proliferation, apoptosis, and epigenetic signals, (vi) Controlled pro-inflammatory signaling with anti-inflammatory feedback. In vitro, MSC1 cells significantly suppressed CRC cell proliferation and metabolic activity versus controls. **Conclusions:** This study provides the first mechanistic map of MSC1’s tumor-suppressive functions in CRC, extending beyond immunomodulation to include metabolic competition, ECM stabilization, and anti-cancer secretome activity. These findings establish MSC1 cells as a novel therapeutic strategy for CRC in cell-based cancer therapies.

## 1. Introduction

Mesenchymal stem cells (MSCs) are multipotent progenitor cells capable of differentiating into adipocytes, chondrocytes, and osteoblasts; they are found in multiple tissues, including bone marrow, adipose tissue, and the umbilical cord. Wharton’s jelly-derived MSCs (WJ-MSCs) are particularly promising for therapeutic use due to their non-invasive isolation, low immunogenicity, high proliferative potential, and strong immunomodulatory functions [1,2].

MSCs have drawn increasing attention in cancer research for their tumor-homing capacity, immunomodulation function, and secretion of bioactive factors [3]. However, studies of MSCs in glioma, Kaposi’s sarcoma, breast, lung, prostate, and colorectal cancers have reported both tumor-suppressive and tumor-promoting effects, reflecting the context-dependent plasticity of MSCs [4].

In colorectal cancer (CRC), the third most diagnosed malignancy and second leading cause of cancer death globally, these divergent effects are especially evident. Some studies report that MSCs inhibit CRC proliferation and invasion via immune activation, paracrine signaling, or exosomal miRNAs [5,6]. Conversely, other reports indicate that MSCs can promote CRC progression by enhancing angiogenesis and epithelial–mesenchymal transition (EMT) and by supporting cancer stemness and metastasis, partly through TGF-β and cancer-associated fibroblast (CAF)-like differentiation [7,8]. These opposing effects are shaped by the tumor microenvironment (TME), a niche marked by hypoxia, high reactive oxygen species (ROS), nutrient deprivation, and inflammatory cytokines [9].

Cancer cells exploit this harsh TME to reprogram surrounding cells, including MSCs, into tumor-supportive phenotypes like CAFs and other pro-tumorigenic stromal populations [10]. In parallel, they actively degrade the extracellular matrix (ECM) to facilitate invasion and metastasis [11]. Stabilizing ECM components, such as laminin-111, which promotes adhesion via α6β1, α6β4, and α2β1 integrins, can suppress motility and maintain tissue integrity [12].

At the metabolic level, normal cells rely on coordinated pathways to meet energy demands and maintain homeostasis. Insulin signaling promotes glucose uptake via GLUT transporters and activates PI3K/AKT pathway to stimulate glycolysis and ATP production. Lipids are also utilized as energy sources and are metabolized via mitochondrial β-oxidation following fatty acid uptake [13]. Phosphoinositide signaling and membrane turnover, including endocytosis and lysosomal recycling, help preserve membrane integrity, protect against oxidative stress, and recover essential bioenergetic resources, such as amino acids, lipids, and nucleotides, for ATP production and biosynthesis. Cancer cells often hijack these pathways to fuel rapid growth and survival in the TME [14].

A key advancement in reconciling MSCs functional duality in cancer came from Waterman et al. [15], who demonstrated that MSCs can be polarized into two distinct phenotypes: MSC1, a pro-inflammatory and tumor-suppressive phenotype induced by low-dose lipopolysaccharide (LPS) via Toll-like receptor 4 (TLR4) over a short period of time, and MSC2, an immunosuppressive and tumor-promoting phenotype induced by poly(I:C). MSC1 cells secrete IL-6, IL-8, CXCL9/10, TNF-α, upregulate MHC-II and co-stimulatory molecules, and enhance immune cell recruitment and activation, mimicking M1-like macrophages. Notably, MSC1 cells have been shown to suppress tumor growth and metastasis in ovarian cancer models by inhibiting angiogenesis and promoting immune surveillance [16]. However, their potential in colorectal cancer remains underexplored.

To address this gap, we investigated the functional programs of the MSC1 phenotype in the TME and validated their tumor-suppressive effects on colorectal cancer cells. We constructed a high-confidence TLR4 signaling network in WJ-MSCs and identified key signaling programs, transcriptional regulators, and novel secreted effectors. We further validated MSC1 cells’ tumor-suppressive activity in CRC through in vitro co-cultures. These findings reveal mechanistic insights into MSC1 cell biology in CRC and demonstrate, for the first time, their tumor-suppressive potential via metabolic competition, ECM stabilization, oxidative stress resistance, and an anti-cancer secretome. 

## 2. Materials and Methods

### 2.1. Acquisition of WJ-MSC Expressed Genes Dataset

A query for “MSC” was performed in the Stemformatics database (v1.9.1) [17], filtering for cell type “Mesenchymal Stromal Cells and Umbilical Cord”, and tissue of origin “Umbilical cord”. We selected the Martins et al. dataset [18] (Dataset ID: 6393, originating from GEO accession GSE51869), which provides author-supplied, pre-processed log_2_ expression values from microarray-based transcriptomic data of MSCs derived from the umbilical cord across 11 samples under physiological conditions. No further normalization was applied by our group. From these values, only Wharton’s jelly-derived MSCs (WJ-MSCs) were retained, and genes with log_2_ expression > 2 were deemed “expressed” to reduce background noise, as is standard practice when using microarray data. A secondary analysis with a lower cutoff (log_2_ > 1.5) produced the same core biological themes, validating the robustness of the threshold. Finally, Ensembl IDs of the retained genes were mapped to HGNC symbols using the g:Convert tool in g:Profiler [19], querying against the *Homo sapiens* reference database.

### 2.2. PPI Network Construction and Hub Analysis

A comprehensive literature review (up to May 2025) was conducted to identify proteins involved in signaling pathways downstream of TLR4 activation by LPS. From these, 182 proteins were retained that were also expressed in WJ-MSCs (Section 2.1).

The HGNC symbols of these 182 proteins were submitted to STRING v11 [20] (species Homo sapiens) to retrieve the full protein–protein interaction (PPI) network. We retained only “experiment” and “database” evidence channels with a confidence score ≥ 0.7. To balance network completeness with interpretability, we limited the network to 20 first-shell and 50 second-shell interactors per seed protein. This approach prioritized high-confidence, biologically relevant nodes, while avoiding excessive network expansion that could obscure key signaling pathways. The resulting network was exported as a tab-delimited text file, imported into Cytoscape v3.10.3 [21,22], and visualized using the Compound Spring Embedder layout, with manual refinement for readability.

Hub proteins were identified using the cytoHubba plugin [23] and ranked according to five network topology metrics: Degree, Edge Percolated Component (EPC), Closeness, Bottleneck, and Betweenness. Metric values were normalized using min–max scaling as

(value − min_value)/(max_value − min_value)

to allow for comparative evaluation across metrics.

### 2.3. Functional Enrichment of PPI Network Clusters

Protein clusters within the PPI network were detected using the MCODE plugin in Cytoscape v3.10.3 [24], with the following parameters:

degree cutoff = 2, node score cutoff = 0.2, node density cutoff = 0.1, k-core = 2, maximum depth = 100, with loops, haircuts, and fluffs enabled.

Each cluster’s HGNC symbols were then independently submitted to Metascape (v3.5 accessed 2 April 2025) [25] for enrichment analysis against the *Homo sapiens* background. We downloaded the resulting .zip file reports and parsed enrichments across four annotation categories: biological process, signaling pathways, transcription factors (TFs), and TF target gene sets. Only statistically significant terms (–log_10_(*p*) > 1.3, equivalent to *p* < 0.05) were retained.

To facilitate visualization, enriched terms across all clusters were subsequently pooled, and for each term, the mean percentage of cluster proteins matched was calculated. This allowed for the depiction of shared versus cluster-specific biological processes, while preserving the independent analysis of each cluster.

### 2.4. Subnetwork Analysis of PPI Network

Four key functional subnetworks (cell adhesion and migration, insulin signaling, endocytosis, and membrane localization) were extracted from the full PPI network in Cytoscape v3.10.3.

For the cell adhesion and migration subnetwork, proteins annotated under adhesion- or migration-related enriched terms were used as seed nodes for shortest-paths analyses, including their direct first neighbors. We first performed targeted shortest-path queries from TLR4 for each of the following groups: PKA subunits; PKC isoforms; CKII subunits; laminin-111 subunits; and integrin α6β1, α2β1, and α6β4. These paths delineated an initial laminin–integrin–kinase circuit. Next, we merged all proteins from the individual TLR4→PKA, TLR4→PKC and TLR4→CKII shortest-path results and used this combined set as new seeds for a subsequent shortest-paths query to reconstruct an extended subnetwork of the laminin–integrin–kinase circuit subnetwork.

For the insulin signaling subnetwork, we used proteins annotated under the insulin signaling enriched term as seeds and ran a separate shortest-paths analysis (including first neighbors) from TLR4.

Similarly, for endocytosis and membrane localization subnetworks, proteins associated with endocytosis- or membrane localization-related enriched terms were each used as seed nodes for independent shortest-paths queries.

All shortest-paths analyses employed Cytoscape’s v3.10.3 built-in algorithm. The resulting subnetworks were reviewed and refined manually to ensure biological relevance.

### 2.5. Identification and Classification of TF Target Genes

Each enriched TF target (Section 2.3) was individually queried in the MSigDB database (v2024.1.Hs) [26], and associated target genes were downloaded as JSON files. Genes not expressed in WJ-MSCs (Section 2.1) were excluded.

For the retained TF target genes, subcellular locations and Gene Ontology (GO) terms were retrieved from UniProt (Release 2025_02) [27]. These annotations were manually curated using Microsoft Excel into the following categories:

Location categories:

(a) ECM;
(b) Cell membrane;
(c) Cytoplasm;
(d) Endosome;
(e) Endoplasmic reticulum (ER).

GO term categories:

(a) Cell death and growth inhibition;
(b) Cell contact;
(c) Glucose and insulin signaling;
(d) Metabolism;
(e) Laminins, integrins, and kinases (PKA, PKC, CKII).

Each TF target gene was manually assigned to categories of interest based on the following combination of location and GO term annotations:

- ECM & Cell Death and Growth Inhibition;
- Cell Membrane & Cell Contact and Cell Death and Growth Inhibition;
- ECM and Cell Membrane & Laminins, integrins, and kinases (PKA, PKC, CKII);
- Cell Membrane and Cytoplasm and Endosome and ER & Glucose and insulin signaling;
- Cell Membrane and Cytoplasm and Endosome and ER & Metabolism.

### 2.6. Transcriptional Enrichment Analysis of TF Target Genes

Gene Set Enrichment Analysis (GSEA, version 4.3.3) [28] software, in pre-ranked mode, was used to conduct an analysis on the combined TF target genes from the categories of interest (Section 2.5). The analysis was performed against the MSigDB human collections (v2024) KEGG, Reactome, Hallmark, GO Biological Process, GO Molecular Function, GO Cellular Component, and BioCarta. Gene sets were constrained between 15 and 500 genes, and all input genes were assigned equal ranks (rank = 1), to perform an unweighted enrichment analysis. Additional GSEA parameters were as follows: number of permutations = 1000; remap to gene symbols = remap_only; enrichment statistic = weighted; collapse mode for probe sets = abs_max_of_probes; normalization mode = meandiv; seed for permutation = timestamp; omit features with no symbol match = true. Results were processed in Microsoft Excel, and only enriched gene sets with an FDR q-value < 0.05 were retained for downstream interpretation.

In parallel, over-representation analysis (ORA) was performed for each TF target gene category using the Enrichr web tool [29]. Analyses were run against the human libraries KEGG (2021), WikiPathways (2024), Reactome (2024), Hallmark (2020), GO Biological Process/Molecular Function/Cellular Component (2023), and BioCarta (2016). Enrichr results (tables of terms, *p*-values, and combined score) were downloaded as TSV files and imported into Microsoft Excel for filtering. Only terms with a *p*-value < 0.05 were retained, and the remaining terms were ranked according to Enrichr’s combined score for downstream interpretation.

### 2.7. WJ-MSC Isolation

Wharton’s jelly-derived mesenchymal stromal cells (WJ-MSCs) were isolated from human umbilical cords obtained from women giving birth, following the procurement of signed informed consent and ethical approval from the Committee for Bioethics and Ethics of the School of Medicine of Aristotle University of Thessaloniki (Protocol Number 4.120). Umbilical cords were processed within 24 h of delivery under sterile conditions.

The umbilical vein and arteries were carefully removed, and the Wharton’s jelly tissue was disinfected with betadine, washed with Dulbecco’s phosphate-buffered saline (PBS) (Biowest, Cat# L0615, Nuaillé, France), minced into small fragments, and enzymatically digested in 0.3% collagenase type I (Worthington Biochemical Corp., Cat# LS004197, Lakewood, NJ, USA), 0.03% hyaluronidase (Worthington Biochemical Corp., Cat# LS002592), 1% penicillin/streptomycin (Biowest, Cat# L0018), and 0.5% gentamycin (Biowest, Cat# L0012) in PBS under gentle shaking (70 rpm) at 37 °C for 3 h, until ~90% tissue dissociation occurred. The resulting suspension was filtered through a 100 µm cell strainer (Corning Inc., Cat# 352360, Corning, NY, USA) to obtain a single-cell suspension and centrifuged at 700× *g* for 15 min at 20 °C. Cells were resuspended in culture medium DMEM (Biowest, Cat# L0104) with 10% fetal bovine serum (FBS, Biowest, Cat# S181H), 1% penicillin/streptomycin (Biowest, Cat# L0018), and 0.5% gentamycin (Biowest, Cat# L0012). Approximately 2 × 10⁶ cells were plated in T75 tissue culture flasks, while the remaining cells were cryopreserved in aliquots into cryovials with 10% DMSO (PanReac Química SLU, Cat#A3672, Castellar del Vallès, Spain).

### 2.8. WJ-MSC and RKO Cell Cultures

The cells were maintained at 37 °C in a humidified atmosphere with 5% CO_2_. The culture medium was high-glucose Dulbecco’s Modified Eagle Medium (DMEM, Biowest, Cat# L0103), supplemented with 10% fetal bovine serum (FBS, Biowest, Cat# S1810) and 1% penicillin/streptomycin. The medium was changed every 2–3 days. When the cultures reached 70–80% confluency, the cells were passaged using 1× trypsin-EDTA (Biowest, Cat# X0930) for 6 min at 37 °C (1 mL for MSCs, 3 mL for RKO). Then, the cells were centrifuged at 1200 rpm for 6 min at 21 °C, resuspended in fresh culture medium, and plated. Only MSCs from passage 4 or below were used for the experiments. The MSC identity was confirmed by adherence to plastic, and positive CD90/CD105 expression was assessed by flow cytometry.

For cryopreservation, cell pellets were resuspended in freezing medium consisting of 90% FBS and 10% DMSO, and 500 μL was aliquoted into sterile 2 mL cryogenic vials (Greiner Bio-One, Cat# 122263, Kremsmünster, Austria). The vials were gradually cooled to −80 °C using a freezing container (Mr. Frosty) and subsequently transferred to liquid nitrogen for long-term storage.

### 2.9. MSC1 Cell Polarization via TLR4 Activation with LPS

Lipopolysaccharide (LPS, from *Escherichia coli* O55:B5, Sigma-Aldrich, Cat# 93572-42-0, St. Louis, MO, USA) was serially diluted in sterile 0.9% NaCl (normal saline) to prepare the polarizing solution. To induce MSC1 cell polarization, MSCs were washed with PBS and incubated for 1 h with culture medium containing 10 ng/mL LPS at 37 °C, as described by Waterman et al. (2010) [15]. After polarization, the cells were thoroughly washed with PBS and cultured in fresh medium. MSC1 cells were used for assays the following day. Successful MSC1 cell polarization was confirmed by quantitative reverse transcription polymerase chain reaction (qRT-PCR) analysis showing upregulation of IL6 and IL8 transcripts following LPS treatment.

### 2.10. MTT Assays of MSC1 and RKO Co-Cultures

WJ-MSCs were plated in equal quantities in 96-well plates and cultured for 3 days. Then, they were polarized to MSC1 with 10 ng/mL LPS, as described above. For the controls, MSCs were treated with culture medium containing an equivalent volume of normal saline (vehicle control). The next day, 5000 RKO cells were seeded into each well containing either polarized MSC1 or control MSCs. Parallel conditions were also set up using MSC1-conditioned medium (MSC1-CM) or control MSC-conditioned medium (MSC-CM).

At 24 and 48 h, the cell viability of the RKOs was assessed by adding 150 μL of MTT solution (0.5 mg/mL MTT in culture medium; Thermo Fisher Scientific, Cat# M6494, Waltham, MA, USA) to each well, followed by incubation for 4 h at 37 °C. After incubation, the MTT solution was removed, 100 μL of DMSO was added to dissolve the formazan crystals under gentle shaking for 10 min, and the absorbance was measured using a μQuant microplate reader (BioTek, Gen5, Winooski, VT, USA). All conditions were tested in triplicate, and six biological replicates were used.

Because the MTT assay quantifies mitochondrial dehydrogenase activity, absorbance serves as a proxy for metabolic activity. Therefore, reductions in RKO absorbance were interpreted as decreased metabolic output and viability, reflecting the tumor-suppressive effects of MSC1 or MSC1-CM.

Raw optical density (OD) values were calculated as:

(570 nm − blank) − (630 nm − blank)

Relative cell viability (%) was ∂ as:

(mean OD of experimental condition/mean OD of control) × 100

Standard error propagation for cell viability was calculated as:

Viability (%) × √[(SD_1_/Mean_1_)^2^ + (SD_2_/Mean_2_)^2^]

The normality of the OD distributions was verified using the Shapiro–Wilk test. Pairwise two-tailed *t*-tests (Student’s or Welch’s, as dictated by the variance ratio) were applied to the raw OD values for each comparison. A separate *t*-test was run for each group pair. Statistical significance was set at *p* < 0.05 (*), *p* < 0.01 (**), and Cohen’s d was calculated to estimate the effect size. Because each experimental group is normalized to its own control (e.g., RKO in conditioned-medium assays), it is expected that some groups will exceed 100% when the control shows a lower baseline absorbance, which reflects a true relative increase in metabolic activity compared to that of the specific control.

### 2.11. Manual Curation of Anti-Cancer Ligands and Receptors from TF Target Genes

TF target genes belonging to the categories of interest

(a) ECM & Cell Death and Growth Inhibition, and
(b) Cell Membrane and Cell Contact & Cell Death and Growth Inhibition

were manually reviewed for evidence of tumor-suppressive activity. We surveyed all available peer-reviewed literature (via PubMed, Google Scholar, Web of Science, and journal websites) and used Microsoft Excel to compile the findings. Only genes with direct, experimentally supported anti-cancer effects, such as inhibition of cancer cell proliferation, induction of apoptosis, suppression of tumor growth and invasion, or the prevention of MSC transformation into pro-tumorigenic phenotypes, like CAFs, were retained for further analysis.

### 2.12. Data Availability

All datasets generated and analyzed during this study, including the high-confidence TLR4 signaling PPI network and the WJ-MSC TF target gene lists, are publicly available in the Zenodo repository (DOI: https://doi.org/10.5281/zenodo.15540017) [30].

## 3. Results

### 3.1. Core Network Hubs in MSC1 Cells Include SRC, AKT1, ITGB1, and NFKB1

**Figure 1:**
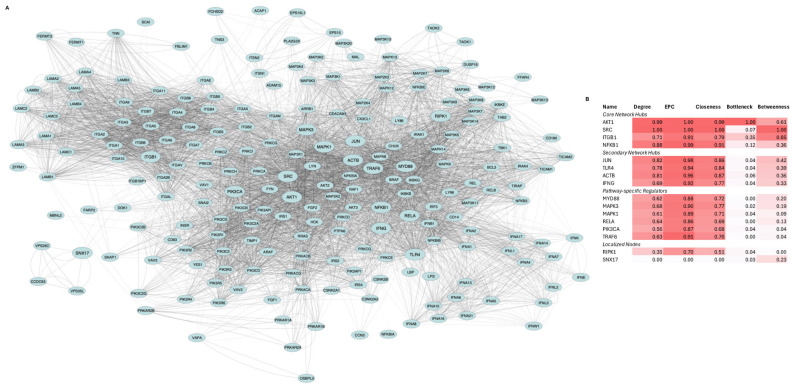
**Network architecture and hub analysis of TLR4-LPS signaling in MSC1 cells. (A). Protein–protein interaction (PPI) network representing signaling responses**. The network consists of 215 proteins (nodes) and 6609 interactions (edges) encompassing downstream signaling cascades, kinase pathways, transcription factor complexes, and extracellular matrix (ECM) interactions. Node size highlights the 16 top-ranked hub proteins, while edge thickness corresponds to interaction confidence. **(B). Heatmap of normalized hub scores (Degree, EPC, Closeness, Bottleneck, Betweenness) for the top-ranked 16 proteins**. Proteins are grouped into core, secondary, pathway-specific regulators, and localized nodes based on centrality and connectivity profiles.

We compiled 182 literature-curated proteins downstream of TLR4-LPS signaling (known to polarize MSCs into the MSC1 phenotype [15]) that are also expressed in WJ-MSCs. Submission of their HGNC symbols to STRING v11.5 yielded a network of 215 nodes and 6609 edges (confidence ≥ 0.7, up to 20 first-shell and 50 s-shell interactors per seed) (Figure 1A). Hub analysis via CytoHubba (Degree, EPC, Closeness, Bottleneck, and Betweenness, min–max normalized) identified 16 top-ranked hubs, which were clustered into four groups and visualized in a heatmap (Figure 1B).

AKT1, SRC, ITGB1, and NFKB1 were the core hubs, defined by both high centrality and high bottleneck scores. JUN, TLR4, ACTB, and IFNG were the secondary hubs, with high centrality but low bottleneck scores. MYD88, TRAF6, MAPK1, MAPK3, PIK3CA, and RELA comprised the third group, exhibiting moderate connectivity. RIPK1 and SNX17 formed the fourth group, with RIPK1 showing moderate centrality and SNX17 exhibiting moderate betweenness.

### 3.2. MSC1 Polarization Activates Kinase Signaling, Immune Modulation, Stress Resistance, ECM Remodeling, and Metabolic Adaptation

**Figure 2:**
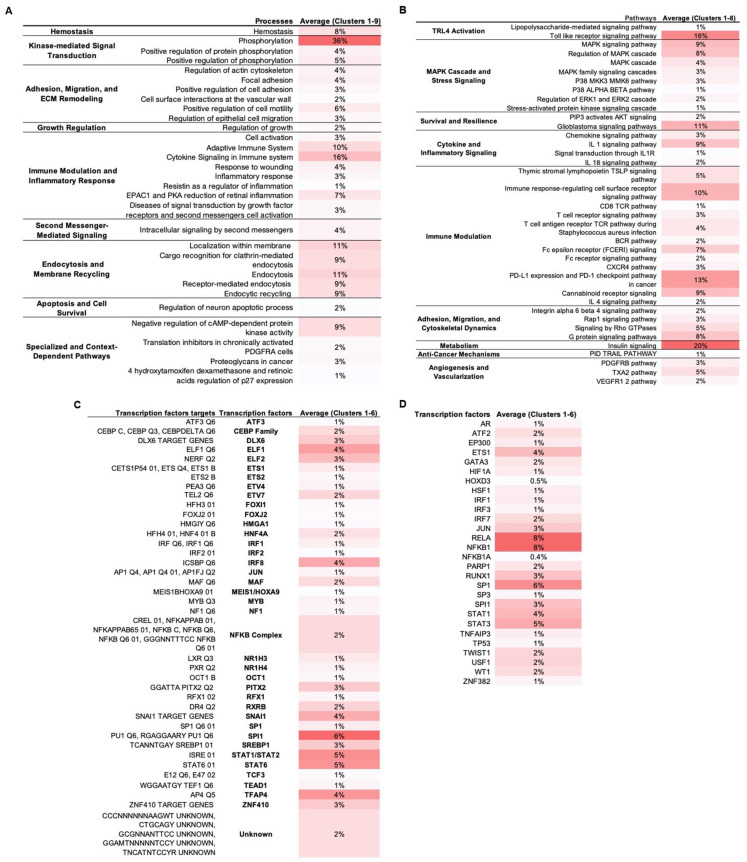
**Functional and transcriptional enrichment of PPI network clusters.** TLR4-LPS downstream network clusters were identified with MCODE plugin in Cytoscape v3.10.3 and enriched in Metascape. Only statistically significant terms were retained (–log_10_(*p*) > 1.3). Values represent percentage of cluster proteins annotated to each term averaged across all clusters, with darker red shading corresponding to higher percentages. **(A). Enriched biological processes. (B). Enriched signaling pathways. (C). Enriched transcription factor targets. (D). Enriched transcription factors.**

MCODE analysis on the TLR4-LPS PPI network in WJ-MSCs yielded nine protein clusters. Each cluster was separately enriched in Metascape (keeping only statistically significant enriched terms with −log_10_(*p*) > 1.3) across biological processes (Figure 2A), signaling pathways (Figure 2B), transcription factors (Figure 2C), and transcription factors targets (Figure 2D), suggesting regulators and downstream effectors in MSC1 cells.

Our analysis indicated five dominant regulatory axes in MSC1 cells: (1) activation of kinase-driven signaling modules, including MAPKs, ERK1/2, and PI3K/AKT; (2) immunomodulation via tightly balanced pro- and anti-inflammatory signaling and transcriptional control; (3) ECM remodeling, integrin signaling, and cytoskeletal reorganization; (4) survival and stress resistance signaling involving balanced apoptotic control, stemness regulation, epigenetic adaptation, and antioxidant defenses; and (5) metabolic reprogramming through the regulation of glucose, lipid, and fructose uptake, as well as insulin signaling and energy homeostasis.

#### 3.2.1. Activation of MAPKs, PI3K/AKT, and Kinase Signaling Modules

Phosphorylation was the top-enriched biological process (36%), with “positive regulation of phosphorylation” term annotated at 5% (Figure 2A). At the pathway level, MAPK sub-pathways (p38, ERK1/2, SAPK/JNK) and secondary-messengers terms, including cAMP protein kinase signaling, were enriched (Figure 2B). Also, PI3K/AKT signaling and the glioblastoma pathway, which involves these axes, were enriched.

#### 3.2.2. Immunomodulation via Balanced Inflammatory and Anti-Inflammatory Signaling and Transcription

Immune activation and pro-inflammatory terms were highly enriched. In biological processes, “cytokine signaling in immune system” was the most enriched (16%), followed by “adaptive immune system (10%)” (Figure 2A). At the pathway level, we observed enrichment of the pro-inflammatory signals (IL-1, IL-1R, IL-18) and the broader immunomodulatory pathways, including TSLP, TCR, BCR, FCERI, CXCR4, and the cannabinoid receptor, along with immunosuppressive PD-L1/PD-1 and IL-4 signaling (Figure 2B). Transcription factor enrichment included the AP-1 components SPI1 and JUN and the NF-kB subunits NFKB1 and RELA (8%) that mediate pro-inflammatory cytokine secretion, as well as STAT1, which is associated with the M1-like pro-inflammatory signaling (Figure 2C). Negative regulators of inflammation were also enriched, including NFKBIA (IκBα) and TNFAIP3 (A20) that negatively regulate NF-κB; ATF3 and STAT6 that suppress pro-inflammatory responses; and MAF that is associated with anti-inflammatory M2-like states (Figure 2C,D). Also, the interferon-associated factors IRF1/2/3/7/8 and ELF1/ELF2 were enriched.

#### 3.2.3. ECM and Cytoskeletal Remodeling and Integrin Signaling

Multiple processes and signaling pathways related to adhesion, migration, and ECM remodeling were enriched, e.g., “positive regulation of cell motility”, “focal adhesion”, and Rap1, Rho GTPase, G protein, and integrin α6β4 signaling (Figure 2A,B). At the transcriptional level, SP1, a regulator of ECM remodeling; PITX2, involved in MSC migration and differentiation; ETS family members (ETS1, ETS2, ETV4, ETV7), that are known to control adhesion and cytoskeletal reorganization; ZNF410, which modulates ECM composition; HOXD3, that contributes to cytoskeletal reorganization and ECM remodeling; and JUN, that connects cytoskeletal changes to inflammatory signaling, were also enriched. Additionally, endocytosis-related terms like “endocytic recycling” and “localization within membrane” also appeared (Figure 2A).

#### 3.2.4. Stress Resistance and Survival Mechanisms via Balanced Proliferation, Survival, Apoptosis, Stemness, and Epigenetic Remodeling

In biological processes, “hemostasis”, “regulation of growth”, and “regulation of neuron apoptotic process” were enriched (Figure 2A). At the pathway level, signaling related to survival (PIP3/AKT), angiogenesis (VEGFR1/2, PDGFRB, and TXA2), and tumor-suppressive paracrine mechanisms (TRAIL) was enriched (Figure 2B). Also, TFs involved in stress responses were enriched (Figure 2C,D). For example, ATF2 is involved in stress response and apoptosis, WT1 in anti-apoptotic signaling, and TEAD1 in mechanotransduction and survival in stiff tumor environments; HSF1 is a key regulator of the heat shock response for survival under stress, HIF-1A is a master regulator of hypoxia responses, NR1H4 mediates metabolic detoxification and oxidative stress resistance, OCT1 regulates genes involved in oxidative stress adaptation and hypoxia, and TP53 is a guardian of genomic stability and oxidative stress response. Additionally, there was enrichment of TFs involved in stemness (TWIST1, FOXJ2, TCF3, and GATA3), differentiation regulation (DLX6, RUNX1, CEBP, MYB, FOXI1, TFAP4, ETS1, AR, and SP3), and chromatin remodeling (MEIS1/HOXA9, HMGA1, RFX1), as well as of the epigenetic modulator EP300 and the DNA-repair enzyme PARP1.

#### 3.2.5. Metabolic Adaptation: Glucose, Lipid, and Energy Homeostasis

Enrichment analysis showed high enrichment of insulin signaling (20%) (Figure 2B). Also, multiple enriched TFs were associated with metabolism (Figure 2C,D). HIF-1, in addition to regulating hypoxia responses, promotes the expression of glucose transporter GLUT1 and lactate dehydrogenase A (LDHA), shifting metabolism from oxidative phosphorylation (OXPHOS) toward anaerobic glycolysis for adapting to hypoxic conditions. USF1 regulates lipid metabolism, including fatty acid synthesis, cholesterol homeostasis, and glucose–insulin balance. SREBP1, a master regulator of lipogenesis, promotes fatty acid and cholesterol biosynthesis, while NR1H3 enhances cholesterol uptake, and RXRB is involved in retinoic acid signaling.

### 3.3. Subnetwork Mapping Identifies Laminin–Integrin–Kinase Mediated Adhesion, Insulin Signaling, and Membrane Dynamics in MSC1 Cells

**Figure 3:**
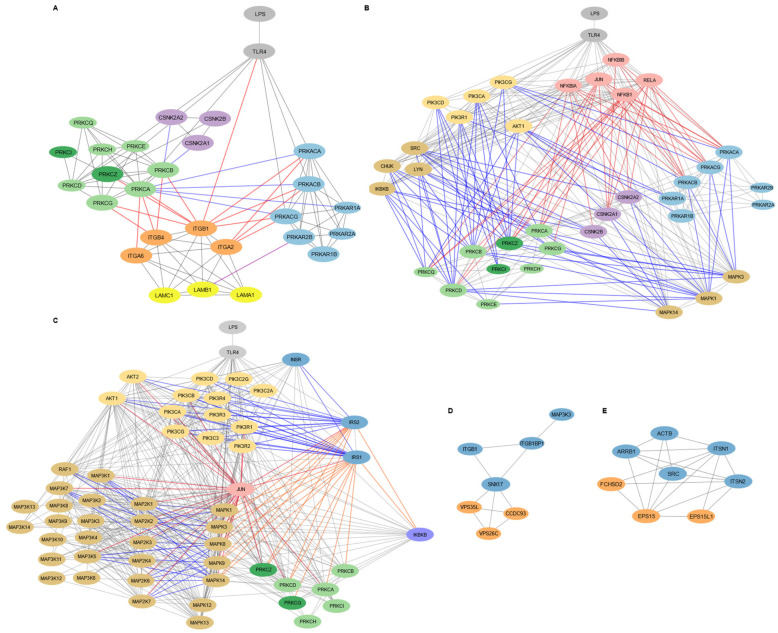
**Targeted subnetwork mapping of LPS-TLR4 signaling in WJ-MSCs.** Subnetworks were reconstructed from shortest path analysis in Cytoscape v3.10.3 seeded with protein hits from the functional enrichment analysis in Metascape (Figure 2). **(A). Laminin–integrin–kinase circuit in cell adhesion and migration subnetwork.** Light gray coloring in nodes shows TLR4 and LPS, light blue indicates PKA subunits, light green highlights DAG-regulated PKC isoforms, dark green indicates PI3K/AKT-regulated PKC isoforms, purple shows CKII subunits, orange indicates integrins (α6β1, α2β1, α6β4), and yellow shows laminin-111 subunits. Gray lines indicate protein–protein interactions, red lines show regulation of integrin activity, purple lines indicate direct PKA-LAMB1 interaction, and blue lines show signaling crosstalk between PKA, PKC, and CKII pathways. Node size highlights proteins that interact directly with integrins and laminins, emphasizing their relevance in adhesion and migration signaling. **(B). Kinase signaling routes to PKA, PKC, and CKII in the adhesion and migration subnetwork.** Light gray nodes display LPS and TLR4, light blue show PKA subunits, light green indicate DAG-regulated PKC isoforms, dark green reflect PI3K/AKT-regulated PKC isoforms, purple indicates CKII subunits, pink shows transcription factors and their inhibitory proteins, pastel mustard reflects kinases involved in PI3K/AKT signaling, and light brown indicates the other kinases. Gray lines reflect general protein–protein interactions, red lines show interactions involving transcription factors, and blue lines represent interactions with kinases. Larger nodes highlight the proteins interacting directly with integrins and laminins or with integrin/laminin-interacting proteins, emphasizing their potential relevance in modulating adhesion and migration signaling downstream of TLR4. **(C). Insulin signaling subnetwork.** Light gray nodes display LPS and TLR4, dark blue shows insulin signaling components, pastel mustard indicates PI3K/AKT signaling proteins, light brown represents MAPK, MAP2K, and MAP3K kinases, light green shows DAG-dependent PKC isoforms, dark green indicates DAG-independent PKC isoforms, pink shows transcription factors, and purple represents other kinases. Gray lines indicate general protein–protein interactions, blue lines show activating interactions, orange lines indicate suppressive interactions, and red lines represent interactions involving transcription factors. **(D). Membrane localization subnetwork.** Proteins in the retromer complex are displayed in orange. **(E). Endocytosis subnetwork.** Blue nodes show fundamental proteins, and orange nodes indicate the other proteins involved.

To refine our understanding of functional and regulatory modules downstream of TLR4 activation in MSC1 cells, we performed subnetwork analyses on the TLR4 downstream PPI network, focusing on subnetworks that regulate cell adhesion and migration, insulin signaling, and membrane dynamics (Figure 3).

#### 3.3.1. Laminin-111/Integrin-Mediated Cell Adhesion and Migration in MSC1 Cells Is Regulated by PKA, PKC, and CKII Signaling

The adhesion and migration subnetwork in MSC1 cells involved signaling by PKA, PKC, and CKII kinases, alongside integrins α2β1, α6β1, and α6β4, and their ligand laminin-111 (Figure 3A). TLR4 directly interacted with catalytic subunits of PKA (PRKACA, PRKACB, PRKACG), PKC isoforms (PRKCA, PRKCD, PRKCZ), and the integrin β1 subunit (ITGB1). PKA catalytic subunits (PRKACA, PRKACB, PRKACG) interacted only with integrin subunits ITGB1 and ITGA2, while PKC isoforms (PRKCA, PRKCB PRKCG, PRKCZ) interacted with integrin subunits ITGA6, ITGB4, and ITGB1. These integrin subunits form the laminin-111 binding integrins α6β1, α2β1, and α6β4. Thus, adhesion and migration in MSC1 cells was organized in tw distinct modules, where PKC targets integrin α6β4 and α6β1, and PKA targets integrin α2β1.

PKA, PKC, and CKII signaling in MSC1 cells appeared to converge through crosstalk mechanisms. CKII subunits (CSNK2A1, CSNK2A2, CSNK2B) interacted with the PKC isoform PRKCB, while the PKC isoform PRKCA interacted with both catalytic and the regulatory PKA subunits (PRKACA, PRKACB, PRKACG, and PRKAR1A). Also, the laminin-111 subunit LAMB1 interacted with PRKAR2B (a regulatory subunit of PKA), anchoring PKA at the ECM surface.

Key inflammatory and signaling kinases also occupy central positions in the adhesion/migration subnetwork (Figure 3B). The NF-κB components RELA and NFKB1, their inhibitors NFKBIA and NFKBIB, and JUN each connect to integrin-interacting PKA catalytic subunits and PKC isoforms, including the non-integrin-binding PRKCQ. The PI3K subunits (PIK3CA, PIK3CD, PIK3CG, PIK3R1) and AKT1 interacted with PKA, PKC, and the CKII catalytic subunits, particularly those targeting laminin-111-binding integrins. PIK3R1 also interacted with non-integrin–binding PRKCD and the PKA regulatory subunit PRKAR1A, while AKT1 interacted with the PKA regulatory subunit PRKAR1B. Additionally, the PKC and CKII subunits interacted with kinases SRC, LYN, CHUK, IKBKB, and MAPKs (MAPK1, MAPK3, MAPK14), with MAPK1 and MAPK3 also uniquely interacting with the PKA subunits.

#### 3.3.2. Insulin Signaling Pathway in MSC1 Cells Intersects with MAPK and Inflammatory Networks

The insulin subnetwork involved the canonical insulin signaling components INSR, IRS1, IRS2, the PI3K isoforms (PIK3CA, PIK3CB, PIK3CD, PIK3CG, PIK3C2A, PIK3C2G, PIK3C3, PIK3R1, PIK3R2, PIK3R3, PIK3R4), AKT1, AKT2, and the downstream effectors, including MAP2Ks/MAP3Ks (MAP2K1, MAP2K2, MAP2K3, MAP2K6, MAP2K7, MAP3K1, MAP3K5, MAP3K7, RAF1) (Figure 3C). It also included the inhibitory regulators of the canonical insulin signaling, including the PKC isoforms (PRKCA, PRKCB, PRKCD, PRKCZ, PRKCQ), the MAPKs (MAPK1/ERK1, MAPK3/ERK2, MAPK8/JNK1, MAPK9/JNK2, MAPK14/p38), and IKBKB. Also, JUN interacted with IRS1/2, AKT1/2, the MAPK regulators (MAPK1, MAPK3, MAPK8, MAPK9, MAPK14), the upstream MAP2Ks and MAP3Ks, and the PKC isoform PRKCZ.

#### 3.3.3. Endocytosis and Membrane Localization Subnetworks in MSC1 Cells

The membrane-localization subnetwork included SNX17, VPS35L, VPS26C, CCDC93, MAP3K3, ITGB1, and ITGB1BP1 (Figure 3D). SNX17 emerged as a central hub, interacting with both the integrin β1 subunit (ITGB1) and its adaptor protein ITGB1BP1, as well as with the retromer components VPS35L, VPS26C, and CCDC93. ITGB1BP1 directly connected ITGB1 to SNX17, while also interacting with MAPK3. MAP3K3 interacted only with ITGB1BP1.

In parallel, the endocytosis subnetwork included EPS15, EPS15L1, FCHSD2, SRC, ARRB1, ACTB, ITSN1, and ITSN2 (Figure 3E). EPS15 and EPS15L1 function in cargo recognition and recruitment to clathrin-coated pits, facilitating the internalization of membrane proteins and signaling receptors. FCHSD2, in conjunction with scaffold proteins ITSN1 and ITSN2, connected this process to actin cytoskeleton remodeling (ACTB), enabling efficient vesicle formation and intracellular trafficking. These proteins also interacted with SRC, a kinase known to regulate cytoskeletal and endocytic processes, and ARRB1 (beta-arrestin 1), a multifunctional adaptor involved in receptor internalization.

### 3.4. Functional Enrichment of MSC1 Cells TF Target Genes Highlights Metabolic Reprogramming and Tumor-Suppressive Programs

The downstream target genes of each enriched TF target (Figure 2C) were retrieved from MSigDB and filtered for their expression in WJ-MSCs. UniProt annotations for the subcellular localization and GO terms were collected for each target gene and used to assign them to one of five functional group: (1) ECM & Cell Death and Growth Inhibition, (2) Cell Membrane & Cell Contact and Cell Death and Growth Inhibition, (3) ECM and Cell Membrane & Laminins and Integrins and Kinases (PKA, PKC, CKII), (4) Cell Membrane and Cytoplasm, and Endosome and ER & Glucose and Insulin, and (5) Cell Membrane and Cytoplasm and Endosome and ER & Metabolism.

We then ran Gene Set Enrichment Analysis (GSEA), in the pre-tanked, unweighted mode, using the combined target genes of all functional groups and performed over-representation analysis (ORA) separately for each group.

#### 3.4.1. Balanced Regulation of Growth, Apoptosis, and Survival in MSC1 Cells via TGF-β, Kinase, and Growth Factor Signaling

Terms associated with kinase signaling and secondary messengers, such as “Diacylglycerol Dependent Serine Threonine Kinase Activity” and “P38 MAPK pathway”, as well as terms related to TGF-β and fibroblast growth factor (FGF) signaling and EMT (leading genes *FGFR4, FGFR3, FGFR2, FGFR1, THBS1*), were enriched in the GSEA analysis (Table 1). Also, terms related to apoptosis and the regulation of cell growth and cell death, like “negative regulation of cell growth” and “negative regulation of anoikis”, an ECM-detachment-induced apoptosis, were enriched in the cell membrane, cell contact, cell death, and growth inhibition category of the ORA analysis (Table 2). Additionally, terms related to signaling axes that are commonly hijacked by cancer cells, such as terms related to ALK, TROP2, and TOB1, which is a growth-suppressive factor, and EGFR and ERBB (leading genes *DAB2IP* and *PTPRJ*) were enriched in both analyses.

#### 3.4.2. MSC1-Mediated Proliferation Suppression and Immune Modulation by Secretion of TNFs, NO, Cytokines, and Direct Cell–Cell Contact

Immune modulatory and inflammatory terms, such as “Fc Gamma R Mediated Phagocytosis” and “Immune Infiltration in Pancreatic Cancer”, were enriched in both GSEA and ORA analyses. Also, in the ECM, cell death, and growth inhibition category of the ORA analysis, there were enrichments of terms related to growth inhibition and apoptosis (Table 2). For example, key enrichments included “Negative Regulation of Smooth Muscle Cell Proliferation” (leading genes *IL10*, *BMP4*, *BMP2*, *IGFBP5*, *TGFB3*, *PTEN*, *IL12B*, and *APOE*), “TNFs Bind Their Physiological Receptors” (leading genes *TNFRSF6B*, *TNFSF14*, *TNFSF15*, *TNFSF13*, *LTA*, *TNFSF11*, *TNFRSF11B*, *TNFRSF1B*, and *TNFSF13B*), and “Regulation of Nitric Oxide Mediated Signal Transduction”. Moreover, in the cell membrane, cell contact, cell death, and growth inhibition category of the ORA analysis, terms related to direct cell–cell contact, such as “cadherin binding” and “contact inhibition”, were enriched.

#### 3.4.3. ECM Remodeling Promotes Tumor-Suppressive Matrix Stabilization via Integrins and MSC1 Cell Migration via Cytoskeletal Reorganization

Enrichment of ECM remodeling was prominent in both analyses. In GSEA, enriched terms included “integrin complex”, with leading genes involving multiple integrin subunits, and “regulation of extracellular matrix organization” (Table 1). In ORA, in the ECM, cell death, and growth inhibition category, enrichments included terms related to ECM and vascular interactions, such as “Platelet Mediated Interactions With Vascular And Circulating Cells”, “Regulation Of Collagen Biosynthetic Process” (Table 2). Also, in the cell membrane, cell contact, cell death, and growth inhibition category of the ORA, terms associated with cytoskeletal dynamics and cell migration were enriched.

#### 3.4.4. Stress Resistance via ROS Signaling, Antioxidant Adaptation, and Epigenetic Modification

ORA revealed stress-response enrichments (Table 2). In the cell membrane, cell contact, cell death, and growth inhibition category, the term “Negative Regulation Of Cellular Response To Hypoxia”, with leading gene *ENO1*, was enriched. Also, in the cell membrane, cytoplasm, endosome, ER, and Metabolism category, enriched terms included “SOS-mediated Signaling” and “mRNA Protein and Metabolite Induction Pathway By Cyclosporin A” with leading genes *SLC3A2*, *SLC7A11*, *NFE2L2*, and *ATF4*. Notably, SLC3A2 and SLC7A11 form the cystine/glutamate antiporter system xc-, essential for glutathione (GSH) biosynthesis, which detoxifies reactive oxygen species (ROS) and protects against ferroptosis. NFE2L2 (NRF2) is a master transcriptional regulator of antioxidant defenses, and ATF4 orchestrates the integrated stress response (ISR), critical for adaptation to oxidative and nutrient stress. Also, there was enrichment of EMT regulators, such as SNAI1, and tumor suppressors, like ZNF382 and NF1 (Figure 2C,D).

#### 3.4.5. Metabolic Adaptation via Glucose, Lipid, and Amino Acid Pathways

In the ORA analysis, multiple metabolism-related terms were enriched (Table 2). In the cell membrane, cytoplasm, endosome, ER, glucose, and insulin category, enriched terms included “Insulin Receptor Signaling Pathway” and “Negative Regulation of Insulin Receptor Signaling Pathway”, as well as “PI3K/AKT/mTOR Signaling”. Also, enriched terms included “Positive Regulation of Glucose Import” and “Glucose Transmembrane Transport”, with leading genes *SLC2A14*, *SORT1*, *SLC2A12*, *SLC2A1*, *SLC2A3*, *SLC2A4*, *SLC2A5*, and *SLC5A3*.

Moreover, in the cell membrane, cytoplasm, endosome, ER, and metabolism category, enrichment of the lipid metabolism and lipid signaling pathways was evident. Enriched terms included “Lysosphingolipid and LPA Receptors”, “Arachidonate Production From DAG”, “Regulation Of Long-Chain Fatty Acid Import Across Plasma Membrane”, with leading genes *AKT2*, *ACSL5*, *IRS2*, and *THBS1*, and “Glycerophospholipid Metabolic Process”, with phospholipase-related genes *PLA2G4D*, *PLCG1*, and *GDPD3*. Also, enriched terms related to the regulation of phospholipase C and inositol signaling were observed. Additionally, “Fructose Metabolism”, with leading genes *TKFC*, *ALDH1A1*, *SORD*, and *ALDOB*, and “Positive Regulation Of Protein Catabolic Process In The Vacuole” were enriched.

### 3.5. MSC1 Co-Culture and Secretome Reduce Colorectal Cancer Cell Viability by Suppressing Metabolic Activity

**Figure 4:**
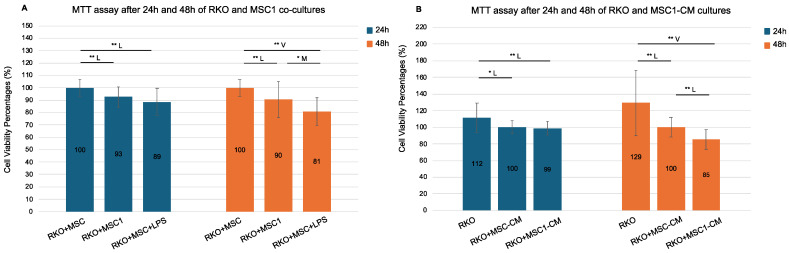
**MTT assay following 24 h and 48 h RKO co-cultures with MSC1 cells and MSC1 conditioned media (CM).** RKO cells were cultured for 24 h and 48 h with control MSCs (RKO+MSC), MSC1 (RKO+MSC1), LPS-treated MSCs (RKO+MSC+LPS), and their respective conditioned media (RKO+MSC-CM, RKO+MSC1-CM). MSC1 phenotype was induced by treating MSCs with 10 ng/mL LPS for 1 h. Bars show mean cell viability percentage ± propagated standard error (n = 6 biological replicates). Values were normalized against their respective controls: RKO+MSC was used as the 100% control for both RKO+MSC1 and RKO+MSC+LPS, while RKO+MSC-CM served as the 100% control for both RKO+MSC1-CM and RKO. Values > 100% reflect higher MTT absorbance relative to the designated control. Pairwise two-tailed *t*-tests were performed separately for each bracketed comparison (Student’s or Welch’s, depending on variance equality). Horizontal brackets denote tested pairs. Significance is annotated as * *p* < 0.05 (95% confidence) and ** *p* < 0.01 (99% confidence). Effect size categories (Cohen’s d) are indicated by a letter above each bracket: M = medium (0.5–0.8), L = large (0.8–1.3), and V = very large (>1.3). Pairs without a horizontal bracket were not significant. **(A). RKO co-cultures with MSC1 and MSC cells. (B). RKO mono-cultures with MSC1-CM and MSC-CM.**

To further explore and validate the MSC1-mediated suppression of cancer cell growth through metabolic competition and secreted ligands, we co-cultured MSC1 cells with the colorectal cancer cell line RKO and treated RKO cultures with MSC1-conditioned media (MSC1-CM) for 24 and 48 h, assessing metabolic activity via MTT assays (Figure 4).

RKO cells co-cultured with MSC1 cells exhibited significantly reduced viability compared to control RKO+MSC co-cultures, both after 24 h (93%) and 48 h (90%), with statistical significance at 99% confidence (*p* = 0.002 and *p* = 0.006, respectively) and large effect sizes (Cohen’s d = −0.99 and −0.82). Therefore, MSC1 cells reduced the cell viability of RKO after 24 h and 48 h. Notably, RKO co-cultures with MSCs in the presence of 10 ng/mL LPS (RKO+MSC+LPS) also reduced cell viability compared to the control RKO+MSC co-cultures (89% at 24 h and 81% at 48 h, *p* = 0.000 for both) with very large effect sizes (d = −1.25 and −2.01). Viability was also significantly reduced in RKO+MSC+LPS co-cultures versus RKO+MSC1 co-cultures only at 48 h (*p* = 0.043, d = −0.75).

Additionally, RKO monocultures treated with MSC1-conditioned media (MSC1-CM) also showed time-dependent reductions in viability. After 24 h, there was no significant difference between RKO+MSC1-CM and control RKO monocultures treated with MSC-CM (RKO+MSC-CM) (99% versus 100%, *p* = 0.514), but by 48 h, RKO+MSC1-CM cultures exhibited significantly reduced viability (85%) compared to the RKO+MSC-CM controls (100%), with a large effect size (*p* = 0.000, d = −1.23). Cell viability of RKO+MSC1-CM was also reduced both after 24 h and 48 h compared to RKO monocultures cultured in culture medium alone (RKO) at 99% confidence (*p* = 0.006 and *p* = 0.001, respectively) with large and very large effect sizes (d = −0.93 and d = −1.51, respectively). Therefore, the culture medium of MSC1 cells reduced the cell viability of RKO both after 24 h and 48 h. Interestingly, even RKO monocultures treated with MSC-CM (RKO+MSC-CM) showed significantly lower viability compared to RKO monocultures (*p* < 0.05 at 24 h and *p* < 0.01 at 48 h, respectively). Yet, the observed reduction of RKO cell viability was stronger for MSC1-CM than MSC-CM.

### 3.6. MSC1 Cells Express a Tumor-Suppressive Ligand and Receptor Profile

We manually examined the TF Target Genes specifically within the functional categories “ECM and Cell Death and Growth Inhibition” and “Cell Membrane, Cell Contact, Cell Death and Growth Inhibition” for known anti-cancer roles in CRC and other tumor contexts, and compiled a set of ligands and receptors with putative tumor-suppressive function in MSC1 cells (Table 3).

#### 3.6.1. Apoptotic TNF Superfamily Members (TRAIL, LIGHT, TL1A, LTA) and Decoy Receptors

We identified multiple TNF superfamily members with direct apoptotic effects on cancer cells, including *TNFSF10 (TRAIL)*, *TNFSF14 (LIGHT)*, *TNFSF15 (TL1A)*, and *LTA*. TRAIL selectively binds to DR4 and DR5 receptors on cancer cells, triggering apoptosis via caspase activation while sparing normal cells [31]. LIGHT (TNFSF14) binds to TNFRSF14 (HVEM) and LTβR on cancer cells, promoting apoptosis. Additionally, it can activate NK cells and dendritic cells to elicit a tumor-specific T-cell immune response [32]. However, LIGHT may also exhibit tumor-promoting effects in specific contexts such as colorectal cancer liver metastases [33]. TL1A (TNFSF15) binds to death receptor 3 (DR3) on cancer cells, where it induces apoptosis, and also acts on immune cells to promote anti-angiogenesis and macrophage M1 polarization [34,35]. However, TL1A has also been implicated in promoting metastasis and EMT through the TGF-β/Smad3 pathway in CRC [36]. LTA, although primarily secreted by lymphocytes, has been shown to promote tumor suppression via LTβR signaling in cancer cells [37,38]. Notably, we also identified decoy receptors TNFRSF11B and TNFRSF6B, which bind TRAIL and TL1A/LIGHT, respectively, as well as TNFRSF1B (TNFR2), which is a receptor for TNF-α.

#### 3.6.2. BMPs (BMP2, BMP4, BMP7) and Inhibins for Growth Suppression

Bone marrow proteins (BMPs) *BMP2*, *BMP4*, and *BMP7*, bone marrow protein receptors (BMPRs) *BMPR2* and *ACVR1*, as well as the inhibins *INHA* and *INHBA*, were identified among the WJ-MSC enriched TF target genes. BMP2, BMP4, and BMP7 are canonical growth-inhibitory ligands known to suppress tumor progression by inducing apoptosis and inhibiting EMT in CRC and other cancers [39,40,41]. INHA and INHBA, inhibit activin signaling by competing for activin receptor binding, thereby reducing proliferation in cancer cells [42].

#### 3.6.3. Novel Putative Ligands and Receptors with Anti-Cancer Potential: SLIT3, CCN3, DCN, DNASE1L3, SULF1, THBS1, PTEN, OAS1, P2RX4, CDHR2, PTPRs, and DAB2IP

In addition to the above well-characterized proteins, novel or less-characterized tumor-suppressive ligands and proteins were identified. SLIT3 suppresses cancer cell migration and angiogenesis via ROBO signaling [43]. CCN3 is anti-proliferative and anti-invasive in CRC, with reduced expression linked to progression [44]. DCN binds and inhibits EGFR and MET, reducing proliferation and angiogenesis [45]. DNASE1L3 degrades extracellular DNA (ecDNA), counteracting immunosuppressive neutrophil extracellular traps in the TME [46]. SULF1 inhibits HSPG-mediated growth factor signaling, limiting tumor growth [47]. THBS1 inhibits angiogenesis and induces apoptosis, although it may play dual roles in the TME [48]. PTEN, while mostly intracellular, can be secreted and internalized by tumor cells to inhibit PI3K/AKT signaling [49]. OAS1, while primarily cytosolic, degrades dsRNA and can trigger stress-induced apoptosis if internalized by CRC cells [50].

Receptor-level regulators included P2RX4 (a purinergic receptor activated by extracellular ATP to fuel MSCs by causing ion fluxes) [51], CDHR2 (promotes cell adhesion and limits migration), and phosphatases PTPRJ and PTPRH, which attenuate EGFR signaling [52], along with DAB2IP, an intracellular suppressor of Ras/MAPK and PI3K/AKT signaling.

## 4. Discussion

In this study, we used an integrative systems biology approach, combining PPI network analysis, transcriptional and functional enrichment, and experimental validation to uncover how MSC1 cells execute their tumor-suppressive function. Our results reveal a multifaceted tumor-suppressive program orchestrated by MSC1 cells that involves coordinated kinase signaling cascades, metabolic reprogramming, paracrine ligand secretion, direct cell–cell interactions, ECM remodeling, cytoskeletal reorganization, stress adaptation, immune modulation, transcriptional control, and epigenetic regulation. Collectively, these features may equip MSC1 cells with the capacity to migrate to sites of inflammation, adapt to the TME, suppress cancer cell growth, and resist conversion into tumor-supportive CAFs.

PPI network analysis identified AKT1, SRC, ITGB1, and NFKB1 as central hubs, JUN, TLR4, ACTB, and IFNG as secondary hubs, MYD88, TRAF6, MAPK1, MAPK3, PIK3CA, and RELA as key signaling axes, and RIPK1 and SNX17 as localized nodes (Figure 1). The prominence of AKT1, SRC, NFKB1, RELA, and JUN suggested that kinases AKT1 and SRC and the NFkB/AP-1 transcriptional modules drive signal transduction and metabolic reprogramming in MSC1 cells. The high centrality of ITGB1 and ACTB implied critical roles in integrin adhesion, ECM interactions, and cytoskeletal dynamics while IFNG’s hub status raises the hypothesis of autocrine/paracrine interferon signaling. Moderate betweenness of SNX17 points to a linking role in endocytic tracking. Moderate centrality of MYD88, TRAF6, MAPK1/3, RIPK1, and PIK3CA aligns with their known roles in canonical TLR4 pathways and validated the structure of the constructed network. Functional and transcriptional enrichment analyses further supported the activation of MAPK, PI3K/AKT, NFkB, AP-1 complex, and PKA/PKC signaling in MSC1 cells (Figure 2B,C). Our results align with prior studies implicating MAPKs and AKT in MSC resilience [53] and PI3K/AKT, MAPK, AP-1 complex, and NFkB signaling being activated downstream of LPS-induced TLR4 activation [54], but we extend these findings by linking them to MSC1 cells’ tumor-suppressive behavior.

Our functional and transcriptional analysis suggested that maintaining an immunomodulatory balance is a defining feature of MSC1 cells. We observed enrichment of the pro-inflammatory pathways (e.g., TLR, IL-1, IFN, and TCR) and TFs (STAT1, STAT3, SPI1, and IRF1/2/3/7/8), together with anti-inflammatory feedback circuits (PD-L1/PD-1, IL-4, ATF3, STAT6, and TNFAIP3) (Figure 2). This coordinated, biphasic program with built-in feedback likely serves to curb excessive inflammation in the TME and prevent tumor progression. Additionally, enrichment of IRFs, ELF1/2, and ISRE suggests that MSC1 cells engage in type I interferon-driven antiviral and antigen-presentation pathways. Also, enrichment of the Fc receptor pathways, although traditionally restricted to immune cells, raises the hypothesis that MSC1 cells may upregulate these receptors under inflammatory conditions to aid in phagocytosis, apoptotic clearance, or immune coordination, which is consistent with their M1-like phenotype and tumor-suppressive role (Figure 2B and Table 1). These findings are consistent with the MSC1 cell polarization model proposed by Waterman et al. [15], but here we define the biphasic and tightly controlled immunomodulatory signaling in MSC1 cells.

Our functional enrichment, GSEA/ORA, and subnetwork analyses also suggested that ECM remodeling, cytoskeletal reorganization, and laminin-111–integrin signaling are critical to MSC1 cell-mediated tumor suppression. Enrichment of cell adhesion and migration, integrin, cytoskeletal reorganization, and ECM remodeling terms, together with TFs ETS1/2, ETV4/7, HOXD3, SP1, and JUN, suggested that MSC1 cells stabilize the ECM to maintain structural integrity rather than to promote matrix degradation. This dual capability, stabilizing the ECM while retaining migratory capacity, may limit cancer invasion, supported by prior links between ECM stabilization with tumor suppression [55]. Enrichment of angiogenesis-related signaling suggested that MSC1 cells also modulate endothelial behavior, vascular remodeling, and pericyte recruitment to support tissue homeostasis and oxygen delivery within the TME. Moreover, enrichment of cAMP/PKA- and secondary messenger-related terms suggested that MSC1 cells rely on these mechanisms for signal propagation.

Notably, our subnetwork analysis proposed a novel kinase–integrin axis downstream of TLR4. In this model, MSC1 cell adhesion is mediated by laminin-111 and integrins α6β1, α2β1, and α6β4, which are differentially regulated by PKA and PKC (Figure 3A,B). Specifically, PKC targets integrin α6β4 and α6β1, while PKA targets integrin α2β1, for regulation. Direct TLR4-PKA/PKC–integrin interactions raised the hypothesis of a rapid adhesion and migration mechanism, bypassing intermediate adaptors. Further, crosstalk among PKA, PKC, and CKII kinases and key upstream regulators (SRC, LYN, CHUK, IKBKB, MAPKs, and NFkB subunits and inhibitors) suggested a highly coordinated kinase network. In this network, classical PKC isoforms (PRKCA, PRKCB, PRKCG) initiate adhesion via calcium/DAG, PRKCZ sustains long-term adhesion independently of secondary messengers via PI3K signaling, CKII modulates PRKCB activity, and PRKCA regulates PKA catalytic subunits in a calcium-independent manner through its interaction with PRKCZ. The interaction of AKT1 and PIK3R1 with the PKA regulatory subunits (PRKAR1B and PRKAR1A) likely governs PKA localization and activity, while PIK3R1-PRKCD crosstalk may fine-tune PI3K signaling. Finally, the interaction of RELA/NFKB1/JUN and their inhibitors with the PKA/PKC/CKII subunits proposed a tight coupling of inflammatory transcriptional control with kinase-driven adhesion signaling and suggested that these TFs regulate or are regulated by kinase signaling, potentially coordinating transcriptional and post-translational control of adhesion and migration.

Significantly, our network included a direct interaction between the regulatory PKA subunit PRKAR2B and the LAMB1 subunit of laminin-111, suggesting a mechanism by which laminin-111 anchors PKA at the membrane near the integrins (Figure 3). Upon cAMP binding, this anchoring may facilitate the rapid phosphorylation of integrins or ECM molecules to promote adhesion and ECM remodeling. Moreover, this may establish a feedback mechanism whereby PKA regulates its own anchorage and downstream signaling via phosphorylating components of the laminin-111 complex. This interaction aligns with prior reports of ecto-kinase activity by PKA, PKC, and CKII, where these kinases phosphorylate laminin-111 and other ECM components to modulate adhesion dynamics and matrix architecture [56,57,58]. These findings extend our earlier work, in which we mapped the phosphorylation of laminin-111 targeted by PKA, PKC, and CKII within biologically functional interaction sequences, revealing their roles in ECM structure and integrity in both physiological and cancer-associated settings [59,60,61]. To our knowledge, this is the first study to implicate laminin-111-mediated adhesion as a feature of the MSC1 phenotype. Collectively, these results uncover a novel laminin-111–integrin–kinase adhesion circuit in MSC1 cells, which may contribute to tumor suppression by stabilizing the ECM and limiting cancer cell invasion.

Additionally, enrichment of clathrin-mediated endocytosis terms suggested the activation of dynamic membrane turnover to regulate receptor availability and protein homeostasis in MSC1 cells. Our subnetwork analysis proposed a novel endocytosis and membrane localization network in MSC1 cells (Figure 3D,E). In this model, retromer components (VPS35L, VPS26C, and CCDC93) coordinate receptor trafficking back to the cell membrane, MAP3K3 and ITGB1BP1 modulate integrin trafficking and membrane dynamics, and the EPS15/EPS15L1/FCHSD2 endocytic module, together with SRC and ARRB1, coordinate receptor and membrane endocytic internalization coupled to regulatory feedback loops. This process may be mediated in response to extracellular cues, integrating external signals with intracellular trafficking decisions.

Moreover, our analysis suggested another axis of MSC1 cell function that involves balancing proliferation, survival, differentiation, apoptosis, and stemness signaling. Enrichment of terms related to homeostasis, PI3P/AKT signaling, and regulation of growth, apoptosis, and anoikis was observed. Also, survival-promoting TFs (HSF1, TEAD1, and WT1), tumor suppressors (SNAI1 and ZNF382), and regulators of apoptosis (ATF2), stemness (TWIST1, TCF3, and GATA3), and differentiation (DLX6, RUNX1, CEBP, MYB, FOXI1, TFAP4, ETS1, AR, and SP3) were enriched (Figure 2). Enrichment of TGF-β and SMAD signaling suggested that MSC1 cells fine-tune this pathway for ECM remodeling, immune modulation, and lineage flexibility (Table 1 and Table 2) [62]. These enrichments suggested that MSC1 cells activate a transcriptional program to balance survival versus apoptosis and stemness versus differentiation signaling for maintaining cellular homeostasis and self-renewal, inducing controlled differentiation, while suppressing proliferation and lineage commitment. Notably, enrichment of the pro-oncogenic pathways (ERBBs, ALK, FGF, TGF-β, and TROP2), with leading genes such as *DAB2IP*, *PTPRJ*, and *PTPRH*, and EMT-related terms, with leading genes *FGFR4*, *FGFR3*, *FGFR2*, *FGFR1*, *THBS1*, suggested that MSC1 cells downregulate these pathways to avoid CAF formation and EMT, maintaining their tumor-suppressive phenotype in the TME. Stress adaptation through both pro- and anti-apoptotic signaling has previously been observed in WJ-MSCs [63], but here, we propose that MSC1 cells overlay this with a self-limiting layer of signaling control to prevent transformation.

Enrichments of terms related to TRAIL, TNFs, and nitric oxide raised the hypothesis that MSC1 cells secrete paracrine factors that induce oxidative stress and apoptosis, as a direct tumor-suppressive mechanism (Table 1 and Table 2). Enrichment of cadherin-binding and contract-inhibition related terms implied that MSC1 cells engage in direct cell-cell interactions with cancer cells to trigger anti-proliferative and pro-apoptotic effects.

Another axis of MSC1 cell function proposed by our analysis was a robust stress resistance mechanism, involving redox, hypoxia, and stress defenses, at the transcriptional level. Enrichment of TFs HIF1A, TP53, NR1H4, and OCT1 suggested hypoxic and stress resistance. Enrichment of hypoxia-related terms with leading gene ENO1 suggested that MSC1 cells not only deploy hypoxic responses, but also activate feedback mechanisms to prevent tumor-promoting excessive hypoxic signaling. ROS-related enrichments with leading genes ATF4 and NRF2, and the system xc− (SLC3A2/SLC7A11) that regulates glutathione production, protecting against ferroptosis, suggested antioxidant and integrated stress-response reactions. These responses are particularly relevant in the ROS-rich and hypoxic TME, as they may allow MSC1 cells to maintain their viability and function. Moreover, enrichment of TFs involved in epigenetic remodeling (MEIS1, HOXA9, HMGA1, RFX1, PARP1, and EP300) indicated that MSC1 cells may use long-term chromatin reprogramming to maintain cell identity, support stable survival, and suppress oncogenic drift over time.

Metabolic reprogramming was suggested as a hallmark of MSC1 cells. Insulin- and glucose-related enrichments proposed the upregulation of glucose uptake (via GLUT1, GLUT3, and GLUT4) and feedback control mechanisms to avoid hyperactivation and maintain balanced signaling in MSC1 cells (Table 2). PKC, MAPK, and IKBKB inhibitors in the insulin subnetwork (Figure 3C) also suggested a feedback mechanism to avoid overactivation of insulin signaling via kinase-mediated IRS1/2 phosphorylation. Given the suggested activation of PI3K/AKT signaling in MSC1 cells, PI3K/AKT interactions with IRS1/2 in the insulin subnetwork raised the hypotheses of a dual insulin-signaling axis, involving a non-canonical activation axis that bypasses INSR. This dual-mode activation, INSR-dependent and TLR4-driven, may allow MSC1 cells to adapt their metabolic activity dynamically within the TME. HIF-1A enrichment suggested that MSC1 cells shift from oxidative phosphorylation toward glycolysis via upregulation of GLUT1 and LDHA (Figure 2D). Additionally, enrichment of lipid-related terms and TFs (USF1, SREBP1, and NR1H3) suggested a coordinated regulation of lipid and cholesterol metabolism, an increase in lipid uptake, and the usage of the PLC and inositol pathway in MSC1 cells. MSC1 cells may upregulate these axes to rewire lipid metabolism and engage in active membrane remodeling and lipid recycling for energy generation, intracellular signaling, metabolic homeostasis, and antioxidant defense, while avoiding the accumulation of pro-oncogenic lipid intermediates. Enrichment of fructose metabolism suggested that MSC1 cells exploit alternative sugars to meet energy demands when glucose is scarce in the TME. Notably, additional TF target genes identified in our dataset include solute carriers, such as SLC2A12, SLC2A15, SLC2A5, SLC5A3, SLC1A2, SLC38A1, SLC3A2, and SLC7A11 (Table S1), suggesting that MSC1 cells mobilize a broad nutrient transport program for survival and nutrient competition in the TME. Also, the enriched term “upregulation of lysosomal and vacuolar catabolism” implies that MSC1 cells upregulate endocytosis and proteolytic turnover as a metabolic strategy to preserve homeostasis and fuel amino acid pools for protein biosynthesis under stress. This aligns with findings that MSCs adapt metabolically under low oxygen conditions [64], and our study extends this mechanism specifically to the MSC1 phenotype. Together, these enrichments suggested metabolic reprogramming in MSC1 cells to respond and adapt to the challenging conditions of the TME and to sustain their anti-tumorigenic phenotype through nutrient competition with cancer cells, energy regulation, and signaling control.

The metabolic adaptation of MSC1 cells was also supported by the in vitro MTT assays in the MSC1-RKO co-cultures (Figure 4). Given that MTT is a metabolic assay, a reduction in RKO cells’ viability reflects a decrease in their metabolic activity. We showed that MSC1 cells suppressed the metabolism of RKO cells. The MSC1 cells induced suppression of the RKO cells’ metabolism, which may be attributed to nutrient competition of the RKO cells with the MSC1 cells for glucose and lipid resources. Interestingly, MSC1-RKO co-cultures in the presence of LPS showed even greater suppression, possibly because LPS sustains MSC1 cell polarization or re-polarizes newly-proliferated MSCs. This is supported by the presence of *TLR4* among the TF target genes, regulated by SPI1, SREBP1, and ELF1 (Table S2). Interestingly, LPS itself may also directly inhibit RKO proliferation, particularly in wild-type APC CRC cell lines like RKO and Caco-2 [65,66]. Moreover, the MSC1-conditioned medium alone also reduced RKO viability at 48 h, further supporting that the MSC1 secretome contains potent tumor-suppressive signals. Notably, the MSC-conditioned medium alone also reduced RKO viability, to a lesser extent, implying a baseline of modest anti-cancer secretome activity that is enhanced by MSC1 cell polarization. Taken together, these findings demonstrate the direct tumor-suppressive role of MSC1 cells by both direct cell–cell interactions and secreted paracrine factors, independent of immune modulation.

To identify potential mediators of this paracrine suppression, we manually curated candidate secreted and membrane proteins from the TF target genes (Table 3). Based on transcriptional predictions, the MSC1 cells were proposed to upregulate canonical tumor-suppressive ligands, including TRAIL, TL1A, LIGHT, and BMPs, which drive apoptosis and growth arrest in colorectal and other cancers [31,32,33,34,35,36,37,38,39,40,41]. Importantly, the predicted co-expression of decoy receptors (TNFRSF11B and TNFRSF6B) may serve to prevent autocrine apoptosis, a novel mechanism of self-preservation. Similarly, the predicted co-expression of BMPRs (BMPRs BMPR2, and ACVR1) may indicate a mechanism of MSC1 cells to fine-tune BMP signaling, both internally and externally. Also, we identified novel predicted tumor-suppressive ligands (SLIT3, DCN, CCN3, THBS1, INHA and INHBA), proteins (DNASE1L3, SULF1, PTEN, OAS1, and DAB2IP), and receptors (P2RX4, CDHR2, PTPRJ, and PTPRH) in MSC1 cells. Their roles in inhibiting angiogenesis, reducing proliferation, suppressing migration and invasion, preventing CAF formation, and degrading extracellular dsDNA or interfering with receptor-level signal propagation suggested that MSC1 cells actively deplete tumor-supportive signals in the TME. Differentially expressed gene (DEG) analysis of BM-MSCs treated with 10 ng/mL LPS for 4 h (GEO dataset GSE81478) showed the upregulation of DAB2IP, TRAIL, OAS1, TNFRSF11B, and TNFRSF1B (Table S3), confirming their expression downstream of TLR4 activation and supporting the likelihood that these factors are indeed involved in MSC1 cell function [67,68,69,70]. The tumor-suppressive genes that did not show differential expression at the 4 h mark may still be transiently upregulated at the 1 h time point required for MSC1 cell polarization, but may return to baseline by 4 h.

Altogether, our integrative pipeline, combining curated PPI networks, transcriptional and functional enrichment, and in vitro co-culture validation, provides a robust framework to dissect and harness anti-tumor programs in MSCs. Our findings suggest that MSC1 cells exert their tumor-suppressive effects through a coordinated strategy involving survival signaling, stress adaptability, metabolic reprogramming, immunomodulation, ECM remodeling, and a secretome–receptorome axis, with direct anti-cancer activity. This multifaceted mechanism enables MSC1 cells to suppress tumor growth, resist CAF-like transformation, and retain therapeutic potential within the hostile TME. Our findings move beyond the notion of MSC1 cells as merely immunomodulatory and demonstrate their direct tumor-suppressive functions, offering deeper insight into their mechanistic role in the TME and their therapeutic potential in CRC.

This study is limited by its reliance on in vitro validation and computational modeling, which may not reflect the role of MSC1 cells in vivo models. The tumor-suppressive effects of MSC1 cells were evaluated using a single CRC cell line (RKO), and in vivo microenvironmental complexity, including immune cell recruitment, MSC plasticity, and tumor-stroma crosstalk, remains unexplored.

Future studies should assess the persistence and efficacy of MSC1 cells in xenograft and syngeneic tumor models, with emphasis on immunocompetence and phenotype stability. They should also investigate MSC1 cancer cell contact mechanisms and the role of MSC1-derived exosomes. Although our study provides strong mechanistic and functional insights, direct molecular validation of key predicted pathways, such as the confirmation of secreted anti-cancer effectors, adhesion molecules expression, and antioxidant mechanisms, would further strengthen the proposed model. Elucidating these pathways through targeted molecular assays (e.g., proteomics, Western blotting, qPCR) and functional adhesion assays will be critical for fully characterizing MSC1 cell behavior. In addition, incorporating complementary viability and apoptosis assays, such as BrdU incorporation, annexin V/PI staining, or live/dead analysis, could provide further mechanistic detail regarding the anti-cancer effects of MSC1 cells on CRC. Ultimately, understanding how MSC1 cells maintain their anti-cancer properties while avoiding pro-tumorigenic transformation will be essential for translating their potential into safe and effective therapies.

From a translational perspective, our findings suggest that MSC1 cell therapies could serve as adjunctive tools in CRC. Their potential to reshape ECM, suppress cancer metabolism, and secrete pro-apoptotic ligands offers value beyond immunomodulation. However, ensuring sustained MSC1 cell polarization post-injection remains essential for safety and efficacy. Strategies involving the exosome-based delivery of MSC1-derived secretomes may offer a cell-free therapeutic avenue, with reduced risk of transformation or off-target effects.

## Figures and Tables

**Table 1:** **GSEA analysis of combined WJ-MSC-enriched TF target genes in all functional groups.** GSEA run in pre-ranked, unweighted mode against the MSigDB human gene sets (v2024): KEGG, Reactome, Hallmark, GO Biological Process, GO Molecular Function, GO Cellular Component, and BioCarta. Gene-set size was limited to a minimum of 5 and a maximum of 1000. Only enriched pathways with FDR q-value < 0.05 are shown. For each gene-set size, normalized enrichment score (NES), FDR q-value, and leading-edge genes are provided. While NES direction is shown for context, enrichment scores should be interpreted as indicative of over-representation, not expression direction. Due to redundancy across databases, only representative pathways are listed. Complete GSEA results are shown in Table S4.

Enriched Pathway	Size	Nes	FDR q-Value	Leading Edge Genes
**Kinase Signaling and Second Messenger Regulation**				
Diacylglycerol-Dependent Serine Threonine Kinase Activity	9	2.86	0.00	*PRKACA, PRKACB, PRKCB, PRKCD, ATF4, PLA2G5, PLA2G6, PLCB3, PLCB4, PLCB2, ADCY4, ADCY8, EGFR*
Phosphatidylinositol Signaling System	22	2.50	0.00	*PIP4K2A, PIP4K2B, PRKCB, PLCG2, PIP5K1A, PLCG1, ITPK1, PLCB3, PLCB4, PLCB2, PLCD1, DGKA, DGKZ, DGKI, DGKH, ITPR1, PTEN, PIK3C2A*
**Immune Modulation and Inflammation Regulation**				
Fc Epsilon RI Signaling Pathway	19	2.08	0.02	*PRKACB, PRKCB, PLCG2, PLCG1*
Fc Gamma R-Mediated Phagocytosis	26	1.90	0.04	*LYN, ITGA2B, ITGA11, ITGB1, ITGB4, ITGAL, ITGB8, ITGB7, ITGB6, ITGA3, ITGA2, ITGA1, ITGA7, ITGA6, ITGA5*
**Metabolic Adaptation**				
Inositol Phosphate Metabolism	13	2.39	0.00	*PIP4K2A, PIP4K2B, PLCG2, PIP5K1A, PLCG1, ITPK1, PLCB3, PLCB4, PLCB2, PLCD1, PTEN, PIK3C2A*
Chrebp Pathway	5	2.30	0.01	*PRKACA, PRKACB, PLCB2, ADCY4, ADCY8, KCNB1*
**ECM remodeling and Differentiation**				
Integrin Complex	16	2.49	0.02	*GJA1, ITGA2B, ITGA11, ITGB1, ITGB4, ITGB8, ITGB7, ITGB6, DSP, ITGA3, ITGA2, ITGA1, ITGA7, ITGA6, ITGA5*
Regulation of Extracellular Matrix Organization	14	−2.36	0.05	*TGFB2, TGFB1, TGFB3, BMP7, BMP4, BMP2, GSK3B, TGFBR1, TGFBR2*
Regulation of Epithelial to Mesenchymal Transition Involved in Endocardial Cushion Formation	5	−2.37	0.05	*FGFR4, FGFR3, FGFR2, FGFR1, THBS1*
Cellular Component Disassembly	56	−2.47	0.05	*PLEKHA1, VEGFA, INPPL1, PDGFRA, PSEN1, TGFB1, * *TGFB3, BMP7, BMP4, DLG1, FGFR3, FGFR2, FGFR1, TGFBR1, TGFBR2, GREM1, LAMA5*
**Anti-Cancer Mechanisms**				
TGF Beta Signaling Pathway	17	−2.71	0.00	*TGFB2, TGFB1, TGFB3, INHBA, BMP7, BMP4, BMP2, DCN, TGFBR1, TGFBR2, THBS1, MAPK3*
Transforming Growth Factor Beta Receptor Binding	5	−2.38	0.02	*TGFB2, TGFB1, TGFB3, TGFBR1, TGFBR2*
TOB1 Pathway	6	−2.09	0.05	*GRB2, RAC1, HRAS, TGFB2, TGFB1, TGFB3, TGFBR1*
ALK Pathway	12	−2.53	0.00	*WNT4, TGFB1, PTCH1, BMP4, BMP2, DLG1, ILK, GREM1, LAMA5*
Fibroblast Growth Factor Binding	5	−2.43	0.03	*PIK3R4, PIK3R1, ZMPSTE24, CARMIL1, INSR, CTSS, CX3CL1, MAP1LC3A, PLAAT1, PLAAT3, SREBF2, ARHGEF2, DNASE1L3, CAMKK2, LIMA1, VMP1, FAP, IGF1R, TSC2, ADAM15, ADRB2*
P38 MAPK Pathway	7	−2.09	0.04	*TGFB2, TGFB1, TGFB3, TGFBR1, TGFBR2*

**Table 2:** **ORA Analysis of WJ-MSC Enriched TF Target Genes in each functional group.** ORA was performed on the target genes of each functional group separately using Enrichr, against human gene set libraries (KEGG 2021, WikiPathways 2024, Reactome 2024, Hallmark 2020, GO Biological Process 2023, GO Molecular Function 2023, GO Cellular Component 2023, and BioCarta 2016). Only terms with *p* < 0.05 were retained and highest combined scores are shown. Columns list term name, overlap count, *p*-value, combined score, and leading-edge genes. Due to redundancy across databases, only representative pathways are listed. The full ORA results are displayed in Table S5.

Term	Overlap	*p*-Value	Combined Score	Leading Edge Genes
**ECM & Cell Death and Growth Inhibition**				
**TGF-β Signaling**				
Positive Regulation of Pathway-Restricted SMAD Protein Phosphorylation	10/49	0.00	2168	*BMP4, TGFB2, BMP2, TGFB1, GDF15, TGFB3, INHBA, INHA, BMP7, TGFBR2*
**Apoptosis and Cell Death**				
Regulation of Nitric Oxide-Mediated Signal Transduction	3/5	0.00	5069	*THBS1, EGFR, VEGFA*
TNFs Bind Their Physiological Receptors	9/29	0.00	3886	*TNFRSF6B, TNFSF14, TNFSF15, TNFSF13, LTA, TNFSF11, TNFRSF11B, TNFRSF1B, TNFSF13B*
**Cell Growth Inhibition**				
Negative Regulation of Smooth Muscle Cell Proliferation	8/37	0.00	1868	*IL10, BMP4, BMP2, IGFBP5, TGFB3, PTEN, IL12B, APOE*
**Immune Modulation**				
Immune Infiltration in Pancreatic Cancer WP5285	12/39	0.00	5279	*IL10, TGFB2, IL6, LGALS1, TGFB1, TGFB3, IL23A, CCL2, IL12B, LGALS9, MMP9, VEGFA*
**Tissue Repair and ECM Remodeling**				
Platelet Mediated Interactions with Vascular and Circulating Cells	5/17	0.00	1912	*TGFB2, TGFB1, TGFB3, CCL2, PF4*
Regulation of Collagen Biosynthetic Process	5/18	0.00	1734	*BMP4, IL6, TGFB1, TGFB3, WNT4*
**Cell Membrane & Cell Contact and Cell Death and Growth Inhibition**				
**Apoptosis and Cell Death**				
Epithelial Cell Apoptotic Process	2/10	0.00	1982	*BMPR2, DAB2IP*
Negative Regulation of Anoikis	2/16	0.00	1016	*ITGB1, SRC*
**Survival, Proliferation, and Growth Suppression**				
Negative Regulation of Epidermal Growth Factor Receptor Signaling Pathway	3/23	0.00	1639	*DAB2IP, PTPRJ, EGFR*
Negative Regulation of Cell Growth	7/125	0.00	1389	*DDX3X, BMPR2, CDHR2, RACK1, PTPRJ, ENO1, RTN4*
Negative Regulation of ERBB Signaling Pathway	3/18	0.00	2319	*DAB2IP*
TROP2 Regulatory Signaling	4/45	0.00	1321	*ITGB1, SRC, RACK1, EGFR*
**Cell–Cell Contact Signaling**				
Cadherin Binding	24/319	0.00	75,601	*ITGB1, ACVR1, RAB1A, DDX3X, BMPR2, SRC, DAB2IP, PTPRJ, PSEN1, ENO1, PTPRH, FNBP1L, RTN4, EGFR, CD2AP, DLG1, P2RX4, HNRNPK, RUVBL1, RACK1, ITGA6, PKN2, MARK2, EIF4G2*
Contact Inhibition	1/5	0.01	1006	*PTPRJ*
**Cytoskeletal Dynamics and Migration**				
Contractile Actin Filament Bundle Assembly	2/14	0.00	1224	*ITGB1, SRC*
Regulation of Epithelial Cell Migration	4/50	0.00	1143	*BMPR2, SRC, DAB2IP, RTN4*
**Hypoxia**				
Negative Regulation of Cellular Response to Hypoxia	1/5	0.01	1006	*ENO1*
**Cell Membrane and Cytoplasm and Endosome and ER & Metabolism**				
**Lipid Metabolism**				
Regulation of Long-Chain Fatty Acid Import Across Plasma Membrane	4/5	0.00	4686	*AKT2, ACSL5, IRS2, THBS1*
Lysosphingolipid and LPA Receptors	8/14	0.00	2698	*PLPPR1, PLPPR2, LPAR1, S1PR1, LPAR2, S1PR2, S1PR5, S1PR4*
Arachidonate Production from DAG	3/5	0.00	1191	*DAGLA, DAGLB, MGLL*
Glycerophospholipid Metabolic Process	16/62	0.00	999	*PDGFRB, PLA2G4D, PLA2G4B, PLA2G4C, GDE1, PLAAT1, GDPD3, PLA2G6, PLCB3, PNPLA3, PIP5K1A, PLCG1, ENPP6, PLCB2, PLCD1, DGK*
**Lipid Signaling Pathways**				
Positive Regulation of Phospholipase C Activity	12/36	0.00	1206	*PDGFRB, PDGFRA, EDNRA, KIT, LPAR1, LPAR2, HRAS, S1PR4, ESR1, PLCB2, EGFR, FGFR1*
Inositol Lipid-Mediated Signaling	10/33	0.00	838	*PDGFRB, PDGFRA, PLCB3, PLCB4, PLCG2, PLCG1, PLCB2, PLCD1, IGF1R, FGFR1*
**Carbohydrate Metabolism**				
Fructose Metabolism	4/7	0.00	1370	*TKFC, ALDH1A1, SORD, ALDOB*
**Cellular Stress and Defense**				
mRNA Protein and Metabolite Induction Pathway by Cyclosporin A	4/7	0.00	1370	*SLC3A2, SLC7A11, NFE2L2, ATF4*
SOS-Mediated Signaling	4/7	0.00	1370	*IRS1, GRB2, IRS2, HRAS*
Positive Regulation of Protein Catabolic Process in The Vacuole	3/5	0.00	1191	*LRP1, LRP2, LDL*
**Cell Membrane and Cytoplasm, and Endosome and ER & Glucose and Insulin**				
**Insulin Signaling Pathway**				
Insulin Receptor Signaling Pathway	22/47	0.00	11,689	*GSK3B, C2CD5, GSK3A, IRS1, INSR, GAB1, PIK3R3, IRS2, PIK3R2, IDE, PIK3R1, SORBS1, PIK3C2A, SLC39A14, IGF1R, FER, AKT2, GRB2, AP3S1, PTPN2, RHOQ, APPL1*
Negative Regulation of Insulin Receptor Signaling Pathway	11/26	0.00	4338	*GSK3A, SOCS1, IRS1, PRKCB, PRKCD, PIP4K2A, TSC2, PIP4K2B, PRKCQ, TNS2, PTPN2*
IRS Activation	3/5	0.00	2694	*IRS1, INSR, IRS2*
**Glucose Metabolism**				
Positive Regulation of Glucose Import	11/25	0.00	4712	*PRKCI, OCLN, IRS1, CAPN10, AKT2, INSR, IRS2, PIK3R1, SORBS1, RHOQ, APPL1*
Glucose Transmembrane Transport	8/22	0.00	2301	*SLC2A14, SORT1, SLC2A12, SLC2A1, SLC2A3, SLC2A4, SLC2A5, SLC5A3*
**PI3K/AKT/mTOR Signaling Pathway**				*IRS1, INSR, AKT2, IRS2, SORBS1*
PI3K/AKT/mTOR Signaling	8/105	0.00	170	*RAB10, SLC2A4*

**Table 3:** **MSC1 genes with putative direct anti-cancer functions or tumor-suppressive regulatory roles.** Enriched TF target genes were manually curated for published anti-cancer functions, particularly in CRC. The list includes secreted factors, membrane receptors, and intracellular regulators that directly inhibit tumor growth or maintain MSCs’ tumor-suppressive phenotype. Only gene names are shown, without implying confirmed transcriptional regulation.

**Genes encoding proteins with anti-cancer effects on cancer cells**	
Secreted	*TNFSF10 (TRAIL)*, *TNFSF14 (LIGHT)*, *TNFSF15 (TL1A)*, *LTA*, *BMP4*, *BMP7*, *BMP2*, *INHA*, *INHBA*, *SLIT3*, *CCN3*, *DCN*, *DNASE1L3*, *OAS1*, *SULF1*, *THBS1*, *PTEN*
Receptors	*TNFRSF1B (TNFR2)*, *TNFRSF11B*, *TNFRSF6B (DcR3), BMPR2*, *ACVR1*, *P2RX4*, *CDHR2*
**Genes encoding proteins that mediate MSC apoptosis and growth suppression**	
Receptors	*PTPRJ*, *PTPRH*
Intracellular	*DAB2IP*

## Data Availability

All datasets generated and analyzed during this study, including the high-confidence TLR4 signaling PPI network and the WJ-MSC TF target gene lists, are publicly available in the Zenodo repository (DOI: https://doi.org/10.5281/zenodo.15540017) [30].

## Supplementary Materials

The following supporting information can be downloaded at: https://www.mdpi.com/article/10.3390/biomedicines13061503/s1, Table S1. Metabolic genes among WJ-MSC enriched TF target genes downstream of TLR4 activation. Genes names were retrieved from GSEA (MSigDB) using the enriched TF targets from the LPS-induced TLR4 downstream PPI network (Figure 2). Only gene names are shown to provide a concise overview of metabolic involvement, without implying confirmed transcriptional regulation. Table S2. Genes of interest among WJ-MSC enriched TF target genes downstream of TLR4 activation. Twenty-four key genes of interest are listed, including kinases (PKA, PKC, CKI), laminin-111 subunits (LAMA1, LAMC1), TLR4, and TFs (e.g., NFKB1, JUN, RELA). Genes were retrieved from GSEA (MSigDB) using the enriched TF targets from the LPS-induced TLR4 downstream PPI network (Figure 2). Only gene names, TF target motif groups, and their corresponding enriched TFs are shown, without implying confirmed transcriptional regulation. Table S3. Differentially expressed tumor-suppressive TF target genes in BM-MSCs after 4 h LPS (10 ng/mL) treatment (GSE81478). Bone marrow MSCs were profiled via GEO database [67,68] dataset GSE81478 [69,70] and analyzed on the iDEP platform [71] (FDR = 0.2, fold-change ≥ 1). Only significantly upregulated or downregulated TF target genes (from the WJ-MSC PPI network downstream of TLR4 activation) are listed. Table S4. Complete table of GSEA analysis of WJ-MSC enriched TF target genes in all functional groups. GSEA run in pre-ranked, unweighted mode against the MSigDB human gene sets (v2024): KEGG, Reactome, Hallmark, GO Biological Process, GO Molecular Function, GO Cellular Component, and BioCarta. Gene-set size was limited to a minimum of 5 and a maximum of 1000. Only enriched pathways with an FDR q-value < 0.05 are shown. For each gene-set size, normalized enrichment score (NES), FDR q-value, and leading-edge genes are provided. While NES direction is shown for context, enrichment scores should be interpreted as indicative of over-representation, not expression direction. Table S5. Complete ORA analysis of WJ-MSC enriched TF target genes in each functional group. ORA was performed on the target genes of each functional group separately using Enrichr against human gene set libraries (KEGG 2021, WikiPathways 2024, Reactome 2024, Hallmark 2020, GO Biological Process 2023, GO Molecular Function 2023, GO Cellular Component 2023, and BioCarta 2016). Only terms with *p* < 0.05 were retained and highest combined scores are shown. The columns list term name, overlap count, *p*-value, combined score, and leading-edge genes.

## Abbreviations

The following abbreviations are used in this manuscript:

MSCs	Mesenchymal stem cells
MSC1	Mesenchymal stem cells polarized to MSC1 phenotype
CRC	Colorectal cancer
LPS	Lipopolysaccharide
TLR4	Toll-like receptor 4
PPI	Protein–protein interaction
WJ-MSCs	Wharton’s jelly mesenchymal stem cells
EMT	Epithelial–mesenchymal transition
CAF	Cancer-associated fibroblast
ECM	Extracellular matrix
EPC	Edge percolated component
TFs	Transcription factors
TF targets	Transcription factor targets
TF target genes	Transcription factor target genes
GSEA	Gene set enrichment analysis
ORA	Over-representation analysis
qRT-PCR	Quantitative reverse transcription polymerase chain reaction
MSC1-CM	MSC1-conditioned medium
MSC-CM	MSC-conditioned medium
OD	Raw optical density
LDHA	Lactate dehydrogenase A
OXPHOS	oxidative phosphorylation
NO	Nitric oxide
ROS	Reactive oxygen species
IRS	Integrated stress response
TNF	Tumor necrosis factor
ecDNA	Extracellular DNA
TME	Tumor microenvironment
BMPs	Bone marrow proteins
BMPRs	Bone marrow protein receptors
