# Supplementary material for: MSC1 Cells Suppress Colorectal Cancer Cell Growth via Metabolic Reprogramming, Laminin–Integrin Adhesion Signaling, Oxidative Stress Resistance, and a Tumor-Suppressive Secretome
Source: Biomedicines. 2025 Jun 19;13(6):1503. doi: 10.3390/biomedicines13061503 (PMC12191268; doi:10.3390/biomedicines13061503)
Supplement: Supplementary file 1 [file biomedicines-13-01503-s001.zip › Table_S1.pdf]

Table S1. Metabolic Genes Among WJ-MSC Enriched TF Target Genes Downstream of TLR4 Activation. Genes names were retrieved from GSEA (MSigDB) using the enriched TF Targets from LPS-induced TLR4 downstream PPI network (Figure 2). Only gene names are shown to provide a concise overview of metabolic involvement without implying confirmed transcriptional regulation.

Category	Protein Names
Glucose Metabolism	*SLC2A1 (GLUT1), SLC2A4 (GLUT4), SLC2A3 (GLUT3), SLC2A12, SLC2A14, INSR, IRS1, IRS2, SORT1, SORBS1, RAB10, VAMP2, GAPDH, HK2, GYS1, PYGL*
Lipid Metabolism	*SLC2A5, SLC5A3, SLC5A11, ACSL5, THBS1, LPL, PLPPR1, PLPPR2, LPAR1, LPAR2, S1PR1, S1PR2, S1PR5, DAGLA, DAGLB, MGLL, PLA2G4D, PLA2G4B, PLA2G4C, GDE1, GDPD3, GDPD5, ENPP6, CHKA, PLCB2, PLCB3, PLCG1, PLCD1, PIP5K1A, PDGFRA, PDGFRB, ALDOA, TKFC, ALDH1A1, SORD*
Protein Metabolism and Redox	*SLC1A2, SLC38A1, SLC3A2, SLC7A11, NFE2L2, ATF4, GRB2, HRAS, LRP1, LRP2, LDLR, MT3*
