# Supplementary material for: MSC1 Cells Suppress Colorectal Cancer Cell Growth via Metabolic Reprogramming, Laminin–Integrin Adhesion Signaling, Oxidative Stress Resistance, and a Tumor-Suppressive Secretome
Source: Biomedicines. 2025 Jun 19;13(6):1503. doi: 10.3390/biomedicines13061503 (PMC12191268; doi:10.3390/biomedicines13061503)
Supplement: Supplementary file 1 [file biomedicines-13-01503-s001.zip › Table_S2.pdf]

**Table S2. Genes of Interest Among WJ-MSC Enriched TF Target Genes Downstream of TLR4 Activation.** Twenty-four key genes of interest are listed, including kinases (PKA, PKC, CKI), laminin-111 subunits (LAMA1, LAMC1), TLR4, and TFs (e.g., NFKB1, JUN, RELA). Genes were retrieved genes were retrieved from GSEA (MSigDB) using the enriched TF Targets from LPS-induced TLR4 downstream PPI network (Figure 2). Only gene names, TF target motif groups, and their corresponding enriched TFs are shown without implying confirmed transcriptional regulation.

| Number | Gene Name | TF Target Count | TF Targets | TFs |
|---|---|---|---|---|
| 1 | *CSNK2A1* | 4 | DLX6 TARGET GENES, CCCNNNNNNAAGWT UNKNOWN, SNAI1 TARGET GENES, CTGCAGY UNKNOWN | DLX6, SNAI1 |
| 2 | *CSNK2B* | 4 | DLX6 TARGET GENES, ZNF410 TARGET GENES, CETS1P54 01, GGAMTNNNNNTCCY UNKNOWN | DLX6, ZNF410, ETS1, |
| 3 | *ITGA2* | 3 | STAT6 01, WGGAATGY TEF1 Q6, E12 Q6 | STAT6, TEAD1, TCF3 |
| 4 | *ITGA6* | 1 | E12 Q6 | TCF3 |
| 5 | *ITGB1* | 1 | WGGAATGY TEF1 Q6 | TEAD1 |
| 6 | *ITGB4* | 6 | AP1 Q4 01, SNAI1 TARGET GENES, NFKB C, WGGAATGY TEF1 Q6, NFKAPPAB 01, E12 Q6 | JUN, SNAI1, NFKB Complex, TEAD1, TCF3 |
| 7 | *JUN* | 4 | ATF3 Q6, CEBP C, CEBPDELTA Q6, CEBP Q3 | ATF3, CEBP Family |
| 8 | *NFKB1* | 3 | ICSBP Q6, ZNF410 TARGET GENES, SNAI1 TARGET GENES | IRF8, ZNF410, SNAI1 |
| 9 | *NFKBIA* | 14 | NFKB Q6, NFKAPPAB65 01, LXR Q3, NF1 Q6, NFKB Q6 01, CREL 01, SNAI1 TARGET GENES, NFKB C, DR4 Q2, CTGCAGY UNKNOWN, CEBP C, GGGNNTTTCC NFKB Q6 01, STAT6 01, HMGIY Q6 | NFKB Complex, NR1H3, NF1, SNAI1, RXRB, CEBP Family, STAT6, HMGA1 |
| 10 | *NFKBIB* | 5 | NFKB Q6, NFKAPPAB65 01, NFKB Q6 01, NFKB C, NFKAPPAB 01 | NFKB Complex |
| 11 | *RELA* | 7 | ELF1 Q6, AP1 Q4 01, DR4 Q2, CEBP C, STAT6 01, SP1 Q6 01, HMGIY Q6 | ELF1, JUN, RXRB, CEBP Family, STAT6, SP1, HMGA1 |
| 12 | *LAMA1* | 4 | NFKAPPAB65 01, CREL 01, GGGNNTTTCC NFKB Q6 01, NFKAPPAB 01 | NFKB Complex |
| 13 | *LAMC1* | 5 | PEA3 Q6, IRF1 Q6, AP1FJ Q2, AP1 Q4 01, AP1 Q4 | ETV4, IRF1, JUN |
| 14 | *PRKACA* | 9 | RFX1 02, AP1FJ Q2, CCCNNNNNNAAGWT UNKNOWN, CETS1P54 01, ISRE 01, PXR Q2, HNF4 01 B, TEL2 Q6, IRF Q6 | RFX1, JUN, ETS1, STAT1/STAT2, NR1H4, HNF4A, ETV7, IRF1 |

| | | | | |
|---|---|---|---|---|
| 15 | *PRKACB* | 5 | PEA3 Q6, TEF Q6, HFH4 01, CETS1P54 01, TEL2 Q6 | ETV4, HNF4A, ETS1, ETV7 |
| 16 | *PRKAR2A* | 1 | NF1 Q6 | NF1 |
| 17 | *PRKCB* | 1 | IRF1 Q6 | IRF1 |
| 18 | *PRKCD* | 4 | NFKB Q6, NFKB Q6 01, NFKB C, HNF4 01 B | NFKB Complex, HNF4A |
| 19 | *PRKCE* | 4 | ZNF410 TARGET GENES, TCANNTGAY SREBP1 01, GGATTA PITX2 Q2, STAT6 01 | ZNF410, SREBP1, PITX2, STAT6 |
| 20 | *PRKCG* | 4 | RFX1 02, LXR Q3, AP4 Q5, CEBP Q3 | RFX1, NR1H3, TFAP4, CEBP Family |
| 21 | *PRKCH* | 1 | SNAI1 TARGET GENES | SNAI1 |
| 22 | *PRKCI* | 3 | DLX6 TARGET GENES, GGAMTNNNNNTCCY UNKNOWN, WGGAATGY TEF1 Q6 | DLX6, TEAD1 |
| 23 | *PRKCQ* | 2 | AP4 Q5, E47 02 | TFAP4, TCF3 |
| 24 | *TLR4* | 4 | RGAGGAARY_PU1_Q6, PU1_Q6, TCANNTGAY_SREBP1_01, ELF1_Q6 | SPI1, SREBP1, ELF1 |
