# Supplementary material for: MSC1 Cells Suppress Colorectal Cancer Cell Growth via Metabolic Reprogramming, Laminin–Integrin Adhesion Signaling, Oxidative Stress Resistance, and a Tumor-Suppressive Secretome
Source: Biomedicines. 2025 Jun 19;13(6):1503. doi: 10.3390/biomedicines13061503 (PMC12191268; doi:10.3390/biomedicines13061503)
Supplement: Supplementary file 1 [file biomedicines-13-01503-s001.zip › Table_S3.pdf]

Table S3. Differentially expressed tumor-suppressive TF target genes in BM-MSCs after 4 h LPS (10 ng/mL) treatment (GSE81478). Bone marrow MSCs were profiled via GEO database [67,68] dataset GSE81478 [69,70] and analyzed on iDEP platform [71] (FDR = 0.2, fold-change ≥ 1). Only significantly upregulated or downregulated TF Target genes (from the WJ-MSC PPI network downstream of TLR4 activation) are listed.

Gene name	Effect
BMP2	Upregulated
BMPR2	Downregulated
DAB2IP	Upregulated
OAS1	Upregulated
TNFSF10 (TRAIL)	Upregulated
TNFRSF11B	Upregulated
TNFRSF1B	Upregulated
