# Supplementary material for: MSC1 Cells Suppress Colorectal Cancer Cell Growth via Metabolic Reprogramming, Laminin–Integrin Adhesion Signaling, Oxidative Stress Resistance, and a Tumor-Suppressive Secretome
Source: Biomedicines. 2025 Jun 19;13(6):1503. doi: 10.3390/biomedicines13061503 (PMC12191268; doi:10.3390/biomedicines13061503)
Supplement: Supplementary file 1 [file biomedicines-13-01503-s001.zip › Table_S4.pdf]

Table S4. Complete table of GSEA Analysis of WJ-MSC Enriched TF Target Genes in all functional groups. GSEA run in in pre-ranked, unweighted mode against the MSigDB against human gene sets (v2024): KEGG, Reactome, Hallmark, GO Biological Process, GO Molecular Function, GO Cellular Component, and BioCarta. Gene set size was limited to a minimum of 5 and a maximum of 1000. Only enriched pathways with FDR q-value < 0.05 are shown. For each gene set size, normalized enrichment score (NES), FDR q-value, and leading-edge genes are provided. While NES direction is shown for context, enrichment scores should be interpreted as indicative of overrepresentation, not expression direction.

Enriched Pathway	Size	Nes	FDR q-value	Leading Edge genes
Kinase Signaling and Second Messenger Regulation				
Kegg Vascular Smooth Muscle Contraction	22	2.77	0.00	*PRKACA, PRKACB, PRKCB, PRKCE, PRKCD, PRKCQ, PLA2G5, PLA2G6, BRAF, PLCB3, PLCB4, KCNMA1, PLCB2, ADCY4, ADCY8*
Gomf Diacylglycerol Dependent Serine Threonine Kinase Activity	9	2.86	0.00	*PRKACA, PRKACB, PRKCB, PRKCD, ATF4, PLA2G5, PLA2G6, PLCB3, PLCB4, PLCB2, ADCY4, ADCY8, EGFR*
Kegg Phosphatidylinositol Signaling System	22	2.50	0.00	*PIP4K2A, PIP4K2B, PRKCB, PLCG2, PIP5K1A, PLCG1, ITPK1, PLCB3, PLCB4, PLCB2, PLCD1, DGKA, DGKZ, DGKI, DGKH, ITPR1, PTEN, PIK3C2A*
Kegg GnRH Signaling Pathway	21	2.37	0.00	*PRKACA, PRKACB, ADCY4, ADCY8, ITGA2B, ITGA11, ITGB1, ITGB4, ITGB8, ITGB7, ITGB6, ITGA3, ITGA2, ITGA1, ITGA7, ITGA6, ITGA5*
Kegg Taste Transduction	7	2.08	0.02	*PIP4K2B, PRKCB, PRKCE, PRKCD, PLA2G6, PLCG2, PIP5K1A, PLCG1, LYN*
Kegg Long Term Potentiation	15	1.97	0.03	*PRKAG1, PRKAG2, ITGA2B, ITGA11, ITGB1, ITGB4, ITGB8, ITGB7, ITGB6, ITGA3, ITGA2, ITGA1, ITGA7, ITGA6, ITGA5*
Immune Modulation and Inflammation Regulation				
Kegg Fc Epsilon RI Signaling Pathway	19	2.08	0.02	*PRKACB, PRKCB, PLCG2, PLCG1*
Kegg Fc Gamma R Mediated Phagocytosis	26	1.90	0.04	*LYN, ITGA2B, ITGA11, ITGB1, ITGB4, ITGAL, ITGB8, ITGB7, ITGB6, ITGA3, ITGA2, ITGA1, ITGA7, ITGA6, ITGA5*
Kegg Vibrio Cholerae Infection	8	1.91	0.04	*PRKCI, PRKCB, PRKCE, PRKCD, PRKD3, PRKD2, PRKCQ, PKN2, PKN1*
Metabolic Adaptation				
Kegg Inositol Phosphate Metabolism	13	2.39	0.00	*PIP4K2A, PIP4K2B, PLCG2, PIP5K1A, PLCG1, ITPK1, PLCB3, PLCB4, PLCB2, PLCD1, PTEN, PIK3C2A*
Biocarta Chrebp Pathway	5	2.30	0.01	*PRKACA, PRKACB, PLCB2, ADCY4, ADCY8, KCNB1*
ECM remodeling and Differentiation				

Gocc Integrin Complex	16	2.49	0.02	*GJA1, ITGA2B, ITGA11, ITGB1, ITGB4, ITGB8, ITGB7, ITGB6, DSP, ITGA3, ITGA2, ITGA1, ITGA7, ITGA6, ITGA5*
Kegg Arrhythmogenic Right Ventricular Cardiomyopathy ARVC	17	2.33	0.00	*AKT2, PRKCB, PRKCE, PRKCD, PLA2G5, PLA2G6, PLCG2, PLCG1, LYN*
Kegg Dilated Cardiomyopathy	21	2.12	0.02	*PRKACB, PRKCB, ATF4, BRAF, PLCB3, PLCB4, PLCB2, ADCY8, GRIA1, ITPR1*
Kegg Hypertrophic Cardiomyopathy Hcm	21	1.87	0.04	*PRKACB, PRKAR2A, PRKAG1, PRKAG2, ACSBG1*
Gobp Regulation Of Extracellular Matrix Organization	14	-2.36	0.05	*TGFB2, TGFB1, TGFB3, BMP7, BMP4, BMP2, GSK3B, TGFBR1, TGFBR2*
Gobp Regulation Of Epithelial To Mesenchymal Transition Involved In Endocardial Cushion Formation	5	-2.37	0.05	*FGFR4, FGFR3, FGFR2, FGFR1, THBS1*
Gobp Cellular Component Disassembly	56	-2.47	0.05	*PLEKHA1, VEGFA, INPPL1, PDGFRA, PSEN1, TGFB1, TGFB3, BMP7, BMP4, DLG1, FGFR3, FGFR2, FGFR1, TGFBR1, TGFBR2, GREM1, LAMA5*
Gobp Mesonephric Tubule Morphogenesis	12	-2.46	0.04	*TNXB, FAP, NID1, TGFB1, IL6, BMP2, FGFR4, LAMC1, TGFBR1, LAMA1, LAMB2*
Gobp Mesonephros Development	17	-2.68	0.03	*PDGFRA, TGFB3, BMP7, BMP4, DLG1, FGFR2, TGFBR1, TGFBR2*
Gobp Skeletal System Morphogenesis	26	-2.40	0.05	*TGFB2, TGFB1, TGFB3, TGFBR1, TGFBR2*
Gobp Embryonic Skeletal System Morphogenesis	8	-2.72	0.03	*WNT4, TGFB1, PTCH1, BMP7, BMP4, BMP2, DLG1, FGFR2, FGFR1, ILK, GREM1, LAMA5*
Anti-Cancer Mechanisms				
Kegg TGF Beta Signaling Pathway	17	-2.71	0.00	*TGFB2, TGFB1, TGFB3, INHBA, BMP7, BMP4, BMP2, DCN, TGFBR1, TGFBR2, THBS1, MAPK3*
Biocarta TGFb Pathway	7	-2.29	0.01	*TGFB2, TGFB1, TGFB3, TGFBR1, TGFBR2, MAPK3*
Gomf Transforming Growth Factor Beta Receptor Binding	5	-2.38	0.02	*TGFB2, TGFB1, TGFB3, TGFBR1, TGFBR2*
Biocarta TOB1 Pathway	6	-2.09	0.05	*GRB2, RAC1, HRAS, TGFB2, TGFB1, TGFB3, TGFBR1*
Biocarta ALK Pathway	12	-2.53	0.00	*WNT4, TGFB1, PTCH1, BMP4, BMP2, DLG1, ILK, GREM1, LAMA5*
Gomf Fibroblast Growth Factor Binding	5	-2.43	0.03	*PIK3R4, PIK3R1, ZMPSTE24, CARMIL1, INSR, CTSS, CX3CL1, MAP1LC3A, PLAAT1, PLAAT3, SREBF2, ARHGEF2, DNASE1L3, CAMKK2, LIMA1, VMP1, FAP, IGF1R, TSC2, ADAM15, ADRB2*
Biocarta P38 MAPK Pathway	7	-2.09	0.04	*TGFB2, TGFB1, TGFB3, TGFBR1, TGFBR2*
