# Supplementary material for: MSC1 Cells Suppress Colorectal Cancer Cell Growth via Metabolic Reprogramming, Laminin–Integrin Adhesion Signaling, Oxidative Stress Resistance, and a Tumor-Suppressive Secretome
Source: Biomedicines. 2025 Jun 19;13(6):1503. doi: 10.3390/biomedicines13061503 (PMC12191268; doi:10.3390/biomedicines13061503)
Supplement: Supplementary file 1 [file biomedicines-13-01503-s001.zip › Table_S5.pdf]

Table S5. Complete ORA Analysis of WJ-MSC Enriched TF Target Genes in each functional group. ORA was performed on the target genes of each functional group separately using Enrichr, against human gene set libraries (KEGG 2021, WikiPathways 2024, Reactome 2024, Hallmark 2020, GO Biological Process 2023, GO Molecular Function 2023, GO Cellular Component 2023, and BioCarta 2016). Only terms with $p < 0.05$ were retained and highest combined scores are shown. The columns list term name, overlap count, p-value, combined score, and leading-edge genes.

Term	Overlap	P-value	Combined Score	Genes
		ECM & Cell Death and Growth Inhibition		
**TGF-β Signaling**				
Regulation of Epithelial to Mesenchymal Transition Involved In Endocardial Cushion Formation	4/7	0.00	5932	*TGFB2, TGFB1, TGFB3, TGFBR2*
Positive Regulation of Pathway-Restricted SMAD Protein Phosphorylation	10/49	0.00	2168	*BMP4, TGFB2, BMP2, TGFB1, GDF15, TGFB3, INHBA, INHA, BMP7, TGFBR2*
Regulation of Pathway-Restricted SMAD Protein Phosphorylation	11/61	0.00	1982	*GREM1, BMP4, TGFB2, BMP2, TGFB1, GDF15, TGFB3, INHBA, INHA, BMP7, TGFBR2*
TGFBR3 Regulates TGF-beta Signaling	3/8	0.00	1781	*TGFB2, TGFB1, TGFBR2*
Type II Transforming Growth Factor Beta Receptor Binding	3/8	0.00	1781	*TGFB2, TGFB1, TGFB3*
**Apoptosis and Cell Death**				
Regulation of Nitric Oxide Mediated Signal Transduction	3/5	0.00	5069	*THBS1, EGFR, VEGFA*
TNFs Bind Their Physiological Receptors	9/29	0.00	3886	*TNFRSF6B, TNFSF14, TNFSF15, TNFSF13, LTA, TNFSF11, TNFRSF11B, TNFRSF1B, TNFSF13B*
Positive Regulation of Tumor Necrosis Factor Superfamily Cytokine Production	14/82	0.00	2401	*APP, WNT5A, PTPRJ, HMGB1, AGER, THBS1, C1QTNF4, IL1A, IL6, OAS1, IL23A, IL12B, LGALS9, PF4*
Positive Regulation of Tumor Necrosis Factor Production	13/78	0.00	2120	*APP, WNT5A, PTPRJ, HMGB1, THBS1, C1QTNF4, IL1A, IL6, OAS1, IL23A, IL12B, LGALS9, PF4*
**Cell Growth Inhibition**				
Negative Regulation of Smooth Muscle Cell Proliferation	8/37	0.00	1868	*IL10, BMP4, BMP2, IGFBP5, TGFB3, PTEN, IL12B, APOE*
**Immune Modulation**				
Immune Infiltration in Pancreatic Cancer WP5285	12/39	0.00	5279	*IL10, TGFB2, IL6, LGALS1, TGFB1, TGFB3, IL23A, CCL2, IL12B, LGALS9, MMP9, VEGFA*

Positive Regulation of Leukocyte Differentiation	3/7	0.00	2310	*LGALS1, HMGB1, LGALS9*
T Cell Modulation In Pancreatic Cancer	9/46	0.00	1807	*IL10, TGFB2, IL6, LGALS1, TGFB1, FAP, TGFB3, LGALS9, VEGFA*
**Tissue Repair and ECM Remodeling**				
Chondrocyte Differentiation	7/29	0.00	1940	*BMP4, WNT10B, BMP2, TGFB1, CCN3, SULF1, FGFR3*
Burn Wound Healing	13/74	0.00	2302	*TGFB2, TGFB3, HMGB1, INHBA, MMP9, DCN, VEGFA, IL6, LGALS1, SFRP2, CCL2, FGFR3, FGFR2*
Platelet Mediated Interactions With Vascular And Circulating Cells	5/17	0.00	1912	*TGFB2, TGFB1, TGFB3, CCL2, PF4*
Regulation Of Collagen Biosynthetic Process	5/18	0.00	1734	*BMP4, IL6, TGFB1, TGFB3, WNT4*
Cell Membrane & Cell Contact and Cell Death and Growth Inhibition				
**Apoptosis and Cell Death**				
Epithelial Cell Apoptotic Process	2/10	0.00	1982	*BMPR2, DAB2IP*
Regulation of Mitochondrial Depolarization	2/10	0.00	1982	*SRC, RACK1*
Negative Regulation of Anoikis	2/16	0.00	1016	*ITGB1, SRC*
BH Domain Binding	1/5	0.01	1006	*RACK1*
BH3 Domain Binding	1/5	0.01	1006	*RACK1*
**Survival, Proliferation, and Growth Suppression**				
Negative Regulation of Epidermal Growth Factor Receptor Signaling Pathway	3/23	0.00	1639	*DAB2IP, PTPRJ, EGFR*
Negative Regulation of Cell Growth	7/125	0.00	1389	*DDX3X, BMPR2, CDHR2, RACK1, PTPRJ, ENO1, RTN4*
CBL mediated ligand-induced downregulation of EGF receptors Homo sapiens h cblPathway	2/8	0.00	2774	*SRC, EGFR*
Negative Regulation of ERBB Signaling Pathway	3/18	0.00	2319	*DAB2IP*
ERBB2 Signaling Pathway	2/13	0.00	1358	*SRC, EGFR*
ERBB2-EGFR Signaling Pathway	1/5	0.01	1006	*EGFR*
BMP Receptor Activity	1/5	0.01	1006	*BMPR2*
TROP2 Regulatory Signaling	4/45	0.00	1321	*ITGB1, SRC, RACK1, EGFR*
Positive Regulation of Protein Kinase B Signaling	6/93	0.00	1380	*ITGB1, P2RX4, SRC, PTPRJ, RTN4, EGFR*
Regulation of Protein Kinase B Signaling	7/136	0.00	1236	*ITGB1, P2RX4, SRC, RACK1, PTPRJ, RTN4, EGFR*
**Cell-cell contact signaling**				

Cadherin Binding	24/319	0.00	75601	*ITGB1, ACVR1, RAB1A, DDX3X, BMPR2, SRC, DAB2IP, PTPRJ, PSEN1, ENO1, PTPRH, FNBP1L, RTN4, EGFR, CD2AP, DLG1, P2RX4, HNRNPK, RUVBL1, RACK1, ITGA6, PKN2, MARK2, EIF4G2*
Contact Inhibition	1/5	0.01	1006	*PTPRJ*
**Cytoskeletal Dynamics and Migration**				
Contractile Actin Filament Bundle Assembly	2/14	0.00	1224	*ITGB1, SRC*
Stress Fiber Assembly	2/14	0.00	1224	*ITGB1, SRC*
Regulation Of Epithelial Cell Migration	4/50	0.00	1143	*BMPR2, SRC, DAB2IP, RTN4*
Positive Regulation of Microglial Cell Migration	1/5	0.01	1006	*P2RX4*
**Hypoxia**				
Negative Regulation Of Cellular Response To Hypoxia	1/5	0.01	1006	*ENO1*
Cell Membrane and Cytoplasm and Endosome and ER & Metabolism				
**Lipid Metabolism**				
Regulation Of Long-Chain Fatty Acid Import Across Plasma Membrane	4/5	0.00	4686	*AKT2, ACSL5, IRS2, THBS1*
Lysosphingolipid and LPA Receptors	8/14	0.00	2698	*PLPPR1, PLPPR2, LPAR1, S1PR1, LPAR2, S1PR2, S1PR5, S1PR4*
Arachidonate Production From DAG	3/5	0.00	1191	*DAGLA, DAGLB, MGLL*
Glycerophospholipid Metabolic Process	16/62	0.00	999	*PDGFRB, PLA2G4D, PLA2G4B, PLA2G4C, GDE1, PLAAT1, GDPD3, PLA2G6, PLCB3, PNPLA3, PIP5K1A, PLCG1, ENPP6, PLCB2, PLCD1, DGKH*
Glycerophospholipid Catabolism	4/7	0.00	1370	*GDE1, GDPD3, ENPP6, GDPD5*
Glycerolipid Catabolic Process	7/18	0.00	963	*PLA2G4D, PLA2G4B, PLA2G4C, GDE1, GDPD3, ENPP6, MGLL*
Choline metabolism in cancer	20/98	0.00	825	*PDGFRB, PDGFRA, CHKA, PLA2G4D, PRKCB, DGKA, PLA2G4B, PLA2G4C, DGKZ, EGFR, MTOR, AKT2, PIP5K1A, GRB2, PLCG1, RAC1, PLPP2, HRAS, DGKI, DGKH*
**Lipid Signaling Pathways**				
Regulation Of Phospholipase C Activity	6/11	0.00	1794	*PDGFRB, PDGFRA, KIT, ESR1, HRAS, FGFR1*
Positive Regulation Of Phospholipase C Activity	12/36	0.00	1206	*PDGFRB, PDGFRA, EDNRA, KIT, LPAR1, LPAR2, HRAS, S1PR4, ESR1, PLCB2, EGFR, FGFR1*
Inositol Lipid-Mediated Signaling	10/33	0.00	838	*PDGFRB, PDGFRA, PLCB3, PLCB4, PLCG2, PLCG1, PLCB2, PLCD1, IGF1R, FGFR1*

**Carbohydrate Metabolism**				
Fructose Metabolism	4/7	0.00	1370	*TKFC, ALDH1A1, SORD, ALDOB*
Fructose Catabolism	3/5	0.00	1191	*TKFC, ALDH1A1, ALDOB*
**Cellular Stress and Defense**				
mRNA Protein and Metabolite Induction Pathway By Cyclosporin A	4/7	0.00	1370	*SLC3A2, SLC7A11, NFE2L2, ATF4*
SOS-mediated Signaling	4/7	0.00	1370	*IRS1, GRB2, IRS2, HRAS*
Positive Regulation Of Protein Catabolic Process In The Vacuole	3/5	0.00	1191	*LRP1, LRP2, LDL*
Cell Membrane and Cytoplasm, and Endosome and ER & Glucose and Insulin				
**Insulin signaling pathway**				
Insulin Receptor Signaling Pathway	22/47	0.00	11689	*GSK3B, C2CD5, GSK3A, IRS1, INSR, GAB1, PIK3R3, IRS2, PIK3R2, IDE, PIK3R1, SORBS1, PIK3C2A, SLC39A14, IGF1R, FER, AKT2, GRB2, AP3S1, PTPN2, RHOQ, APPL1*
Cellular Response to Insulin Stimulus	28/99	0.00	5833	*GSK3B, C2CD5, GSK3A, IRS1, PDE3B, PIK3R3, IRS2, PIK3R2, PIK3R1, IDE, SLC2A4, PIK3C2A, IGF1R, AKT2, AP3S1, APPL1, PRKCI, INSR, GAB1, SORBS1, SLC39A14, RAB10, FER, CAPN10, GRB2, RHOQ, VAMP2, PTPN2*
Insulin Signaling	36/160	0.00	5452	*GSK3B, SNAP25, GSK3A, IRS1, INPPL1, SLC2A1, PIK3R3, IRS2, PIK3R2, PIK3R1, SLC2A4, FOXO3, PIK3C2A, IGF1R, GYS1, SOCS1, AKT2, STX4, HRAS, MAPK3, PRKCI, PRKCB, INSR, PRKCD, GAB1, TSC2, TSC1, FOS, SORBS1, MTOR, MYO1C, PRKCQ, GRB2, SOS1, RHOQ, VAMP2*
Insulin Signaling in Adipocytes Normal Condition	5/8	0.00	5024	*IRS1, AKT2, INSR, SLC2A4, MTOR*
Multiple antiapoptotic pathways from IGF-1R signaling lead to BAD phosphorylation Homo sapiens h IGF1R Pathway	7/13	0.00	4696	*IRS1, GRB2, PIK3R1, SOS1, HRAS, IGF1R, MAPK3*
Negative Regulation of Insulin Receptor Signaling Pathway	11/26	0.00	4338	*GSK3A, SOCS1, IRS1, PRKCB, PRKCD, PIP4K2A, TSC2, PIP4K2B, PRKCQ, TNS2, PTPN2*
IRS Activation	3/5	0.00	2694	*IRS1, INSR, IRS2*
**Glucose metabolism**				
Positive Regulation of Glucose Transmembrane Transport	13/31	0.00	5092	*C2CD5, PRKCI, IRS1, INSR, IRS2, BRAF, PIK3R1, SORBS1, OCLN, CAPN10, AKT2, RHOQ, APPL1*
Positive Regulation of Glucose Import	11/25	0.00	4712	*PRKCI, OCLN, IRS1, CAPN10, AKT2, INSR, IRS2, PIK3R1, SORBS1, RHOQ, APPL1*

Intracellular Glucose Homeostasis	13/43	0.00	2714	*UNC13B, NGFR, KCNB1, PIK3R2, PIK3R1, NR1D1, SLC39A14, HK2, IGF1R, RACK1, LRRC8D, LRRC8A, PRKACA*
Glucose Transmembrane Transport	8/22	0.00	2301	*SLC2A14, SORT1, SLC2A12, SLC2A1, SLC2A3, SLC2A4, SLC2A5, SLC5A3*
Positive Regulation of Glycogen Metabolic Process	5/14	0.00	1389	*IRS1, INSR, AKT2, IRS2, SORBS1*
Insulin-Responsive Compartment	2/5	0.00	720	*RAB10, SLC2A4*
**PI3K/AKT/mTOR Signaling Pathway**				
PI3K/AKT/mTOR Signaling	8/105	0.00	170	*GSK3B, CDKN1B, PRKCB, SLC2A1, TSC2, PIK3R3, GRB2, HRAS*
